# Receptor-interacting protein kinase 2 (RIPK2) profoundly contributes to post-stroke neuroinflammation and behavioral deficits with microglia as unique perpetrators

**DOI:** 10.1186/s12974-023-02907-6

**Published:** 2023-09-30

**Authors:** Jonathan Larochelle, Ryland J. Tishko, Changjun Yang, Yong Ge, Leah T. Phan, Rachel E. Gunraj, Sofia M. Stansbury, Lei Liu, Mansour Mohamadzadeh, Habibeh Khoshbouei, Eduardo Candelario-Jalil

**Affiliations:** 1 Department of Neuroscience, McKnight Brain Institute, University of Florida, 1149 SW Newell Drive, Gainesville, FL 32610 USA; 2 Department of Microbiology, Immunology and Molecular Genetics, University of Texas Health, San Antonio, TX USA

**Keywords:** RIPK2, Neuroinflammation, Ischemic stroke, Microglia, Blood–brain barrier injury

## Abstract

**Background:**

Receptor-interacting protein kinase 2 (RIPK2) is a serine/threonine kinase whose activity propagates inflammatory signaling through its association with pattern recognition receptors (PRRs) and subsequent TAK1, NF-κB, and MAPK pathway activation. After stroke, dead and dying cells release a host of damage-associated molecular patterns (DAMPs) that activate PRRs and initiate a robust inflammatory response. We hypothesize that RIPK2 plays a damaging role in the progression of stroke injury by enhancing the neuroinflammatory response to stroke and that global genetic deletion or microglia-specific conditional deletion of *Ripk2* will be protective following ischemic stroke.

**Methods:**

Adult (3–6 months) male mice were subjected to 45 min of transient middle cerebral artery occlusion (tMCAO) followed by 24 h, 48 h, or 28 days of reperfusion. Aged male and female mice (18–24 months) were subjected to permanent ischemic stroke and sacrificed 48 h later. Infarct volumes were calculated using TTC staining (24–48 h) or Cresyl violet staining (28d). Sensorimotor tests (weight grip, vertical grid, and open field) were performed at indicated timepoints. Blood–brain barrier (BBB) damage, tight junction proteins, matrix metalloproteinase-9 (MMP-9), and neuroinflammatory markers were assessed via immunoblotting, ELISA, immunohistochemistry, and RT-qPCR. Differential gene expression profiles were generated through bulk RNA sequencing and nanoString^®^.

**Results:**

Global genetic deletion of *Ripk2* resulted in decreased infarct sizes and reduced neuroinflammatory markers 24 h after stroke compared to wild-type controls. *Ripk2* global deletion also improved both acute and long-term behavioral outcomes with powerful effects on reducing infarct volume and mortality at 28d post-stroke. Conditional deletion of microglial *Ripk2* (mKO) partially recapitulated our results in global *Ripk2* deficient mice, showing reductive effects on infarct volume and improved behavioral outcomes within 48 h of injury. Finally, bulk transcriptomic profiling and nanoString data demonstrated that *Ripk2* deficiency in microglia decreases genes associated with MAPK and NF-κB signaling, dampening the neuroinflammatory response after stroke injury by reducing immune cell activation and peripheral immune cell invasion.

**Conclusions:**

These results reveal a hitherto unknown role for RIPK2 in the pathogenesis of ischemic stroke injury, with microglia playing a distinct role. This study identifies RIPK2 as a potent propagator of neuroinflammatory signaling, highlighting its potential as a therapeutic target for post-stroke intervention.

**Supplementary Information:**

The online version contains supplementary material available at 10.1186/s12974-023-02907-6.

## Introduction

Globally, stroke is the second leading cause of death and the third leading cause of disability, with an estimated global cost of US $721 billion [1]. Stroke presents as either ischemic stroke (85% of cases) or hemorrhagic stroke (~ 15% of cases). Current treatments for ischemic stroke are critically limited by their short therapeutic window and exclusion criteria, leaving many patients ineligible to receive treatment. Current treatment also does not adequately address one of the main contributors to post-ischemic stroke brain damage: neuroinflammation. In the acute phase of stroke injury, neuroinflammatory processes contribute to worsening outcomes for stroke patients by contributing to blood–brain barrier (BBB) degradation, peripheral immune cell infiltration, and neuronal cell death [2, 3]. Neuroinflammatory processes occur over hours to days following the initial incidence of stroke. While the initial strong inflammatory process contributes to injury, later inflammatory events, characterized by infiltration of monocytes and other immune cells, facilitates debris clearance and the initiation of regenerative processes [2]. By identifying and then modulating neuroinflammatory processes, we may be able to extend the period of therapeutic intervention and improve outcomes for a greater number of stroke patients.

The blockage of cerebral vasculature that occurs in ischemic stroke causes the rapid cell death of neurons in the infarcted region due to the hypoxic and energy-sparse environment [4]. BBB disruption, increased inflammatory cytokine and chemokine production, and necrotic and apoptotic cell death are all hallmarks of stroke pathology that are only made worse as peripheral immune cells begin invading the brain parenchyma, drawn by chemotactic signals released from dead/dying cells and rapidly responding glia [2, 5–7]. This cascade of events eventually expands from the initial site of injury to the surrounding area, termed the penumbra, and without intervention, this once-healthy tissue succumbs to apoptosis and permanent damage. Dampening this initial cascade of neuroinflammatory events is a promising avenue for post-stroke therapy and may protect salvageable brain tissue that may otherwise be lost.

Innate immune cells use pattern recognition receptors (PRRs) to propagate inflammatory signaling, allowing the cells to rapidly respond to dangers such as invasive pathogens or cellular stress and facilitate an inflammatory response. Dead and dying cells release damage-associated molecular patterns (DAMPs), which are recognized by PRRs on immune cells and elicit highly pro-inflammatory effects. As CNS resident immune cells, microglia play a unique immunological role in ischemic stroke, representing the first responders to stroke injury and utilizing PRRs in their response. More recently, ischemic stroke has been associated with gut permeability and the leakage of bacteria and their products from the gut to the systemic circulation [8–15]. Pathogen-associated molecular patterns (PAMPs) also have potent effects on innate PRRs, eliciting robust inflammatory reactions upon their recognition.

Receptor-interacting serine/threonine protein kinase 2 (RIPK2) has been identified as a critical mediator of inflammation by activating multiple pro-inflammatory and cell death pathways. RIPK2 serves as the primary downstream signaling kinase for the nucleotide-binding oligomerization domain (NOD) 1 and NOD2 PRRs [16]. NOD1/2 receptors have been most notably characterized for their roles in recognizing PAMPs; however, more recent evidence suggests that these receptors may play a more prominent role in recognizing DAMPs than was previously thought [16–18]. RIPK2 is involved in both nuclear factor kappa-light-chain-enhancer of activated B cells (NF-κB) activation and the initiation of caspase-mediated apoptosis via the interaction between RIPK2’s caspase activation and recruitment domain (CARD) and caspase-1 [19, 20]. RIPK2 associates with caspase-1 and promotes oxygen–glucose deprivation-induced cell death [21] and is also an upstream regulator of transforming growth-factor-beta-activated kinase-1 (TAK1) [22], the master regulator of mitogen-activated protein kinase (MAPK) pro-inflammatory signaling, whose inhibition improves outcomes for animals after stroke [23–25]. Further proving its role in neuroinflammatory processes, mice globally deficient for the *Ripk2* allele (*Ripk2*^−/−^) were protected from experimental autoimmune encephalomyelitis, a rodent model of multiple sclerosis [26].

In our study, we sought to mechanistically investigate the role of RIPK2 in the progression of stroke injury by studying the effects of stroke in *Ripk2*^−/−^ mice as well as in mice with microglia-specific deletion of *Ripk2* (termed μKO). We hypothesized that both global knockout and conditional knockout of *Ripk2* in microglia would prove beneficial in reducing acute-stage neuroinflammation and preserving BBB integrity, as well as improving behavioral outcomes in these animals after stroke. We utilized both the transient and permanent middle cerebral artery occlusion (MCAO) models of stroke and measured the effect on infarct size, inflammatory markers, and BBB permeability in the acute stages of injury between our different genetic strains. We next assessed the long-term survival and motor function in our global knockout animals after stroke. We induced pMCAO in aged male and female wild type (WT) and *Ripk2*^−/−^ mice and evaluated their anatomical outcomes after stroke. We also performed bulk RNA-sequencing of microglia isolated from WT and *Ripk2*^−/−^ mice after stroke. Finally, we ran a nanoString nCounter® neuroinflammatory PCR panel to assess the differential expression of various neuroinflammatory genes between WT and μKO mice after stroke.

## Materials and methods

### Animals

Three-to-six-month-old (young) male or 18–24-month-old (aged) C57BL/6J male and female mice (Jackson Laboratory) and mice deficient for the *Ripk2* allele (Jackson Laboratory, stock#007017) were used for this study. Microglia-specific *Ripk2* knockout animals (μKO) were generated by crossing *Tmem119*^CreERT2^ mice (Jackson Laboratory, stock# 031820) and *Ripk2*^flox/flox^ (Jackson Laboratory, stock# 413654; strain name: UFL_GET6446_Ripk2_cKO_B6J_F1) mice for two generations to produce *Tmem119*^CreERT2/wt^;*Ripk2*^flox/flox^ (μKO) and *Tmem119*^wt/wt^;*Ripk2*^flox/flox^ (WT), with the WT serving as age- and sex-matched (male) littermate controls. The μKO animals were viable, fertile, and of normal size and exhibited no physical or behavioral abnormalities. To induce conditional deletion, μKO and WT mice received intraperitoneal tamoxifen injections (Cat#T5648, Sigma-Aldrich 75 mg/kg) daily for 5 days. Mice were subjected to stroke following a 7-day tamoxifen washout period.

Mice were housed in a specific pathogen-free facility with a 12-h light/12-h dark cycle and ad libitum access to food and water. All animal experiment procedures were conducted following the NIH Guide for the Care and Use of Laboratory Animals. All procedures were approved by the University of Florida Institutional Animal Care and Use Committee (animal protocol numbers 201907934 and 202200000201). All experiments and analyses were performed by investigators blinded to animal genotypes. The number of animals for each experiment was calculated based on an a priori power analysis. The specific number of animals for each analysis is stated in the figure legends.

### Induction of transient and permanent middle cerebral artery occlusion

Adult mice were subjected to transient focal cerebral ischemia induced by intraluminal occlusion of the right middle cerebral artery MCA for 45 min, as previously described by our group [27]. Briefly, mice were anesthetized with 3% isoflurane, and surgical levels of anesthesia were maintained by inhalation of 1.5–2% isoflurane in medical-grade oxygen. During surgery, body temperature was maintained at 37 °C using a heating pad and temperature regulator with a rectal probe. The right common carotid artery (CCA), external carotid artery (ECA), and internal carotid artery (ICA) were exposed via a midline vertical incision in the anterior neck. A 12-mm-long 6–0 silicone-coated nylon filament (Doccol, Cat#602123) was advanced gently into the ICA approximately 9–10 mm from the carotid bifurcation until mild resistance was felt, and cerebral blood flow (CBF) was reduced by at least 75% of the baseline value, as assessed by laser Doppler flowmetry (Additional file 1). After 45 min of MCA occlusion, the filament was gently retracted to allow reperfusion (confirmed by laser Doppler flowmetry). The skin was closed, anesthesia discontinued, and the mice were allowed to recover in a temperature-controlled chamber. Sham-operated animals received the same surgical procedures except for the MCA occlusion.

Permanent cerebral ischemia was induced by ligation of the left MCA, as described previously [28]. In short, mice were anesthetized with 1.5–2% isoflurane in medical-grade oxygen. The left CCA was exposed and carefully dissected from the vagus nerve, and a 6–0 silk suture was applied to ligate the left CCA permanently. A skin incision was made between the eye and the left ear under a stereomicroscope, and the temporal muscle was retracted to locate the MCA via skull transparency. A small round craniotomy (~ 1–1.5 mm in diameter) was made between the zygomatic arch and the squamosal bone to expose the MCA using a surgical burr (Cat#726066) connected to a battery-operated Ideal Micro-Drill (Cat#726065, Cellpoint Scientific; Gaithersburg, MA, USA). Sterile saline was applied to the target area, and the meninges covering the MCA were carefully removed using forceps. The MCA distal trunk was permanently ligated using an ophthalmic 9–0 silk suture just before the bifurcation between the frontal and parietal branches of the MCA. A clear interruption of blood flow was visually observed. After surgery, mice were allowed to recover in a temperature-controlled chamber. Sham-operated animals received the same surgical procedures except for the MCA ligation.

### Vessel casting and visualization of the cerebrovasculature

To assess variations in the anatomy of cerebral circulation between *Ripk2*^−/−^ and their wild-type littermates, cerebrovascular anatomy was quantitatively assessed via latex vessel casting [29]. Mice were deeply anesthetized with 2.5–3% isoflurane and injected with 50 mg/kg papaverine hydrochloride dissolved in saline through the jugular vein. After 20–30 s, the heart was quickly exposed, and the descending aorta was clamped using hemostatic forceps. The right atrium was opened, and 1 mL of 1000 U/mL heparin was injected transcardially, followed by 3 mL of blue latex rubber solution (Cat#BR80B, Connecticut Vallet Biological Supply Co., Southampton, MA, USA) with a flow rate of 1 mL/min. The brain was then gently removed from the skull and fixed in 4% PFA for 24 h at 4 °C. The dorsal and ventral surfaces were scanned with an HP Scanjet 8300 scanner at 2400 dpi. Anastomoses on the dorsal surface of the hemispheres were localized by tracing the distal branches of the anterior cerebral artery (ACA) and the MCA to the anastomosis points [30]. Adjacent anastomosis points were connected by the line of anastomoses. The distance from the midline to the lines of anastomoses was measured in coronal planes 2, 4, and 6 mm from the frontal pole in photographs taken from the dorsal brain surface using Adobe Photoshop software.

### Measurement of infarct volume

#### TTC staining

2,3,5-Triphenyltetrazolium chloride (TTC) staining was used to measure brain infarction at 24 h or 48 h after stroke, as previously described [29]. Mice were euthanized following tMCAO and were perfused transcardially with ice-cold saline. Brains were harvested, placed in a slicing matrix (Zivic Instruments, Pittsburgh, PA, USA), and sliced into six 1-mm-thick coronal sections. The fourth section starting from the rostral side was dissected into ipsilateral and contralateral cerebral cortices and subcortices. These tissues were immediately frozen in liquid nitrogen for storage at − 80 °C for later processing. The remaining sections were stained with 2% TTC in phosphate-buffered saline (PBS, pH 7.4) at room temperature for 30 min, then were fixed with 4% paraformaldehyde (PFA) in PBS, pH 7.4. The stained sections were laid rostral-side down and scanned at 600 dpi using an HP Scanjet 8300 scanner (Palo Alto, CA, USA) and saved as a JPEG file. The caudal side of the 3^rd^ section was scanned and served as the representative image of the 4th section, as it corresponds to the rostral side of the 4th slice. The infarction volume was calculated by integrating the lesion areas of all six brain slices, corrected for edema [31].

#### Cresyl violet staining

Cresyl violet staining was performed to measure infarct volume at 28d post-tMCAO, as described in our previous work [29]. Mice were euthanized at 28d post-stroke and transcardially perfused with 10 mL of saline containing 5 mM EDTA using a peristaltic pump at a speed of 5 mL/min, followed by 30 mL of 4% PFA in PBS. Brains were collected and post-fixed in 4% PFA for 24 h, then transferred to PBS for storage at 4 °C. Brains were cut coronally into a series of 30-μm-thick sections on a semi-automated vibrating microtome (Compresstome^®^ Model No. VF310-0Z, Precisionary Instruments; Natick, MA, USA). Cortical infarct volume was quantified from infarcted areas in ten brain sections spaced 0.5 mm apart using Cresyl violet staining. Sections were scanned using an Aperio ScanScope^®^ CS system and analyzed with ImageScope Software (Aperio Technologies; Vista, CA, USA). The border between infarcted (dark purple stain) and non-infarcted area (light purple stain) was outlined and quantified using the software. Infarct volume was calculated by subtracting the area of healthy tissue on the ipsilateral hemisphere from the total area of the contralateral hemisphere.

### Microglia isolation and RNA sequencing (RNA-seq)

Due to observed differences in infarct volume between *Ripk2*^+/+^ and *Ripk2*^−/−^ mice, we subjected *Ripk2*^+/+^ mice to 45 min-tMCAO and *Ripk2*^−/−^ mice to 60 min-tMCAO to correct for differences in infarct size and allowed for 24 h of reperfusion.

For isolation of microglia, brains were minced and enzymatically dissociated with 2 mg/ml collagenase D (Cat#11088866001, Roche) and 0.3 mg/ml DNase I (Cat#DN25, Sigma) in RPMI-1640 for 45 min at 37 °C in an incubator (5% CO_2_). The digested tissues were passed through a 21-gauge syringe for 10 times and then a 40 mm cell strainer to generate a single-cell suspension. Subsequently, the cell suspension was centrifuged and resuspended in 30% Percoll layered on 70% Percoll. Following centrifugation, cells were collected from the 70–30% interface. Isolated cells were stained with LIVE/DEAD Fixable Violet Dead Cell Stain Kit (Cat#L34955) and fluorochrome-conjugated antibody to CD45 (Cat#48-0451-82), CD11b (Cat#CD11B01), and CX3CR1 (Cat#46-6099-42) purchased from Thermo Fisher Scientific. Cells were analyzed using a Sony SH800S cell sorter. After exclusion of dead and doublet cells, microglia were identified as CD45^int^ CD11b^+^ CX3CR1^+^ cell population.

For RNA-seq, total RNA was extracted from FACS-sorted microglia using the RNeasy Plus Micro Kit (Cat# 74034, Qiagen, Germantown, MD, USA) and subsequently used for full-length cDNA synthesis using SMART-Seq HT kit (Cat#634439, Takara Bio; San Jose, CA). RNA-seq libraries were prepared, and data were analyzed as described previously [32–34]. Briefly, raw sequence reads were aligned to the mouse reference genomes (GRCm38) using STAR v2.7.5c. DESeq2 was used to determine significantly expressed genes (DEGs) based on the criteria (TPM > 2, FDR < 0.05, fold change > 1.5). Normalized counts (TPM) values were used for principal component analysis (PCA), and heat map plotting in R. Gene set enrichment analysis (GSEA) was conducted using DAVID (https://david.ncifcrf.gov/).

### Conditional knockout confirmation

To confirm genetic deletion of *Ripk2* from microglia, *Tmem119*^wt/wt^;*Ripk2*^flox/flox^ (WT) and *Tmem119*^CreERT2/wt^;*Ripk2*^flox/flox^ (μKO) animals were perfused transcardially with ice-cold HBSS, and brains were quickly collected. Brains were minced and incubated with Accumax (Cat#AM-105, Innovative Cell Technologies; San Diego, CA, USA) for 5 min at room temperature. Enzymatic reaction was stopped by adding ice-cold HBSS. The tissue was further dissociated using 3-ml syringes with 18G and 21G blunt needles, then strained with a 70 mm cell strainer to remove debris. A 30% Percoll solution was used to remove myelin and fatty debris. Microglia were then isolated following the instructions for the MojoSort™ Mouse P2RY12 Selection Kit (Cat#480113, BioLegend; San Diego, CA, USA), and RT-qPCR was utilized to confirm deletion of *Ripk2* from microglia.

### Protein extraction and Western blotting from brain tissue

At 24 h after tMCAO, brains were dissected after transcardiac perfusion with ice-cold saline. The ipsilateral and contralateral cerebral cortex and subcortex were collected and immediately frozen in liquid nitrogen and saved at − 80° for further processing. Tissues were homogenized in radioimmunoprecipitation (RIPA) lysis buffer consisting of 50 mM Tris–HCl (pH 7.4), 150 mM NaCl, 5 mM EDTA, 1 mM EGTA, 1% NP-40, 0.5% sodium deoxycholate and 0.1% SDS plus protease and phosphatase inhibitor cocktails (Cat#78430 and Cat#78428, respectively, Thermo Fischer Scientific; Rockford, IL, USA), and total protein concentration was determined using the Pierce™ BCA assay kit (Cat#23,277, Thermo Scientific; Rockford, IL). An equal amount of protein was separated using 4–20% polyacrylamide gradient gels (BioRad, Hercules, CA, USA) and transferred to nitrocellulose membranes. Membranes were blocked with 5% milk and were incubated overnight at 4 °C with rabbit anti-RIPK2 (1:1000; Cat#4142S, Cell Signaling Technology, Danvers. MA, USA), rat anti-β-Actin (1:5000; Cat#664802, BioLegend; San Diego, CA, USA), rabbit anti-Zona Occludens 1 (ZO-1; 1:1000; Cat#61-7300, Life Technologies; Grand Island, NY, USA), rabbit anti-Occludin (1:1000; Cat#ab167161, Abcam; Cambridge, MA, USA), rabbit anti-MMP-9 (1:500; Cat#sc-6841-R, Santa Cruz Biotechnology; Dallas, TX, USA). After primary antibody incubation, the membranes were washed 3 times with TBST, and incubated with the following secondary antibodies for 1 h at room temperature: goat anti-rabbit IRDye 800CW (1:30,000; Li-Cor; Lincoln, NE, USA), donkey goat anti-rat IRDye 680LT (1:40,000; Li-Cor; Lincoln, NE, USA). Membranes were visualized, and densitometric analysis was performed using the Odyssey infrared scanner and Image Studio 2.0 software (Li-Cor). Unedited blots are included in Additional file 6.

### RNA extraction and quantitative real-time PCR

Total RNA from 50 μL of the total cortical tissue homogenate was isolated using a modified acid guanidinium thiocyanate–phenol–chloroform extraction method [29, 35]. RNA concentration and purity were determined by a Take3 Micro-Volume Plate Reader (BioTek Instruments; Winooski, VT, USA). Quantitative real-time PCR was performed in a total reaction volume of 10 μL using Luna^®^ Universal One-Step RT-qPCR Kit (Cat#E3005, New England BioLabs; Ipswich, MA, USA) following the manufacturer’s protocol. Reactions were performed in 96-well plates run in a BioRad CFX96 Touch Real-Time instrument. Each reaction was performed in duplicate or triplicate, and the relative expression value for each target gene was calculated using the 2^−ΔΔCt^ method after normalization to the housekeeping gene *Ywhaz*. Microglia conditional knockout was confirmed by normalizing to *18 s* and *Cyc1*. The primer sequences used in the reactions can be found in Table 1.

**Table 1:** Gene abbreviations: *Ripk2*, receptor interacting serine/threonine protein kinase 2; *Tnfα*, tumor necrosis factor alpha; *Il1β*, interleukin 1 beta; *Il6*, interleukin 6; *Cxcl1*, C–X–C motif chemokine ligand 1; *Ccl2*, chemokine C–C motif ligand 2; *Mmp9*, matrix metalloproteinase 9; *Ly6g*, Lymphocyte antigen 6 complex locus G6D; *Nos2*, nitric oxide synthase; *Ywhaz*, tyrosine 3-monooxygenase/tryptophan 5-monoogygenase activation protein zeta

Gene	Primer forward sequence	Primer reverse sequence
*Ripk2*	CGCTGCTCGACAGTGAAAGA	TGCCCAAAAATTCAGGCTCAT
*Tnfα*	AGACCCTCACACTCAGATCA	TCTTTGAGATCCATGCCGTTG
*Il1β*	GACCTGTTCTTTGAAGTTGACG	CTCTTGTTGATGTGCTGCTG
*Il6*	AGCCAGAGTCCTTCAGAGA	TCCTTAGCCACTCCTTCTGT
*Cxcl1*	CCAAACCGAAGTCATAGCCA	GTGCCATCAGAGCAGTCT
*Ccl2*	CATCCACGTGTTGGCTCA	AACTACAGCTTCTTTGGGACA
*Mmp9*	GACATAGACGGCATCCAGTATC	GTGGGAGGTATAGTGGGACA
*Ly6g*	TGGACTCTCACAGAAGCAAAG	GCAGAGGTCTTCCTTCCAACA
*Nos2*	GTTCTCAGCCCAACAATACAAGA	GTGGACGGGTCGATGGTCAC
*18s*	CTTAGAGGGACAAGTGGCG	ACGCTGAGCCAGTCAGTGTA
*Cyc1*	CCAAAACCATACCCTAACCCT	CTGCTCACTGGCTACTGTG
*Ywhaz*	TGTTCTAGCCTGTTTCCCCG	ACGATGACGTCAAACGCTTC

### Immunohistochemistry and immunofluorescence

Mice were anesthetized and transcardially perfused, and brains were collected and sectioned as previously described (see *Cresyl violet staining*). IHC was performed following a standard protocol [36] and rabbit anti-Iba1 (1:5000; Cat#019-19741, Wako Bioproducts; Richmond, VA) was the primary antibody. The secondary antibody for IHC was goat anti-rabbit (1:2000; Cat#5450-0010, SeraCare; Gaithersburg, MD, USA), and the immunoreaction was visualized using a 3,3-diaminobenzidine chromogen solution (DAB substrate kit; Vector Laboratories; Newark, CA, USA). The brightfield images were captured by ScanScope CS and analyzed using ImageJ software.

For immunofluorescence, sections were mounted and permeabilized with 1% Triton X-100 for 10 min, and antigen retrieval was performed by incubating with L.A.B. Solution (Polysciences, Inc; Warrington, PA, USA) for 10 min at 60 °C. Sections were blocked for 1 h at 37 °C in 10% normal goat serum (NGS) with 1% Triton X-100 in PBS. Antibody incubations were performed in 5% NGS with 0.3% Triton X-100. The primary antibodies were rabbit anti-RIPK2 (1:200, Cat#GTX28428, GeneTex Inc; Irvine, CA, USA) and guinea pig anti-Iba1 (1:5000, Cat#234308, Synaptic Systems; Göttingen, Germany). The secondary antibodies were AlexaFluor 594 goat anti-guinea pig (1:1000, Cat#A11076, Invitrogen; Carlsbad, CA, USA) and AlexaFluor Plus 488 goat anti-rabbit (1:2000, Cat#A32731, Invitrogen; Carlsbad, CA, USA). Z-stack image acquisition was performed using × 4 dry and × 40 oil immersion objectives on a confocal laser scanning microscope with a Nikon A1 system (Nikon; Tokyo, Japan). Images were processed using NIS Elements.

### Behavioral tests

Animals were subjected to a host of behavioral tests by investigators blinded to genotype. Tests performed include neurological deficit score, open field locomotor activity test, weight grip test, and vertical grid to assess the neurological performance of mice at various timepoints after stroke.

#### Neurological deficit score

The neurological deficits scoring assessment assesses overall neurological abnormalities along with multiple physical deficits in animal studies of stroke [37, 38]. At 24 h after stroke, a neurological deficit score (NDS) was determined for each animal according to six different parameters: body symmetry, gait, circling behavior, front limb symmetry, compulsory circling, and climbing, as previously described [39, 40]. Additional assessments were performed at 48 h, 7d, 14d, and 21d post-stroke. Each test was scored independently by two trained investigators using a 4-point scoring system (0, no deficits; 4 severe deficits). The average score of each mouse from two investigators was used for statistical analysis by nonparametric tests.

#### Open field

The open field test is a reliable behavioral test to assess locomotor and anxiety-like behaviors in mice. Within 1 week prior to stroke (baseline) and at 24 h, 48 h, 7d, 14d, and 21d post-stroke, the spontaneous locomotor activity of mice was measured in an open field paradigm using automated video tracking software (Anymaze software; Stoelting, Wood Dale, IL, USA) as previously described [28]. Mice were individually placed in an open field chamber (40 × 40 × 40 cm) with grey sidewalls and were allowed to freely explore for 10 min. The total distance traveled was used as the indices of motor/exploratory behavior of each animal. The open field arena was thoroughly cleaned with 70% ethanol between tests.

#### Vertical grid test

The vertical grid test is a sensitive test intended to assess animals' neuromuscular strength and motor coordination after stroke [41, 42]. The vertical grid is an open frame apparatus (55 cm H × 8 cm W × 5 cm L) with a wire mesh (0.8 cm × 0.8 cm aperture) on the backside. The grid is placed in a cage filled with soft bedding material. Within 1 week prior to, and 24 h, 48 h, 7d, 14d, and 21d post-tMCAO induction, each mouse was placed at the highest point on the grid facing downward and was allowed to descend the grid into the cage. A blinded investigator recorded the time required for the animal to descend. Animals were subjected to 3 trials with intervals of 30 s between each trial. The average of the three trials constitutes the animal’s score on the test. Animals that failed to descend the grid within 60 s or were unable to maintain a firm grip of the grid and fell were assigned the maximum score of 60 s for that trial.

#### Weight grip test

The weight grip test was performed with minor modifications from a previous study [43], as we have previously reported [44], to assess the muscular strength of the forepaws. Five different weights (weight-1: 16.2 g, weight-2: 30.4 g, weight-3: 44.6 g, weight-4: 58.2 g, and weight-5: 71.4 g) were prepared by attaching a metal mesh to stainless steel lines. The animals were suspended from the middle/base of the tail and allowed to grasp the first weight (weight-1). A timer starts when the mouse successfully grips the weight using its forepaws, then the animal is lifted until the steel links are completely lifted from the bench. The mouse must hold the weight for 3 s for the test to be successful. If the mouse was able to hold weight-1 for 3 s, the investigator proceeded to the next weight in sequential order. If the mouse were to drop a weight in less than 3 s, the test would conclude, and no further weights would be attempted. The mice were permitted three tries to successfully hold the weight for 3 s. A final score was tallied as a sum of the point of each weight that the mouse holds, multiplied by the number of seconds that weight was held. For example, a mouse that held up to weight-5 for 1 s is assigned a score of 1 × 3 + 2 × 3 + 3 × 3 + 4 × 3 + 5 × 1 = 35.

### NanoString nCounter^®^ neuroinflammation panel

We subjected 90 ng of RNA isolated ipsilateral cortex of the 4th 1-mm-thick coronal brain slice (see *TTC staining* for sample collection details) 48 h after stroke to a nanoString^®^ Neuroinflammation panel. Results from the panel were processed in Rosiland (Rosiland; San Diego, CA, USA) to assess the quality and relative expression of inflammatory markers within the panel. Gene expression and pathway profiles were compiled for each group to assess expression levels relative to WT group.

### Statistical analysis

PRISM software (GraphPad V.8) was used to perform the statistical analyses. An independent unpaired Student’s *t* test (parametric) or Mann–Whitney test (nonparametric) was performed to compare two groups. Two-way ANOVA followed by Bonferroni’s post hoc test was used for multiple comparisons. Gehan–Breslow–Wilcoxon test was used to assess survival between genotypes. Values were expressed as mean ± SEM, and a *P* value of less than 0.05 was considered statistically significant. The number of animals per group is clearly stated in the figure legends. All studies were performed by investigators blinded to the genotype of the animals in analyses.

## Results

### Genetic deletion of *Ripk2* protects against acute injury in ischemic stroke

**Fig. 1:**
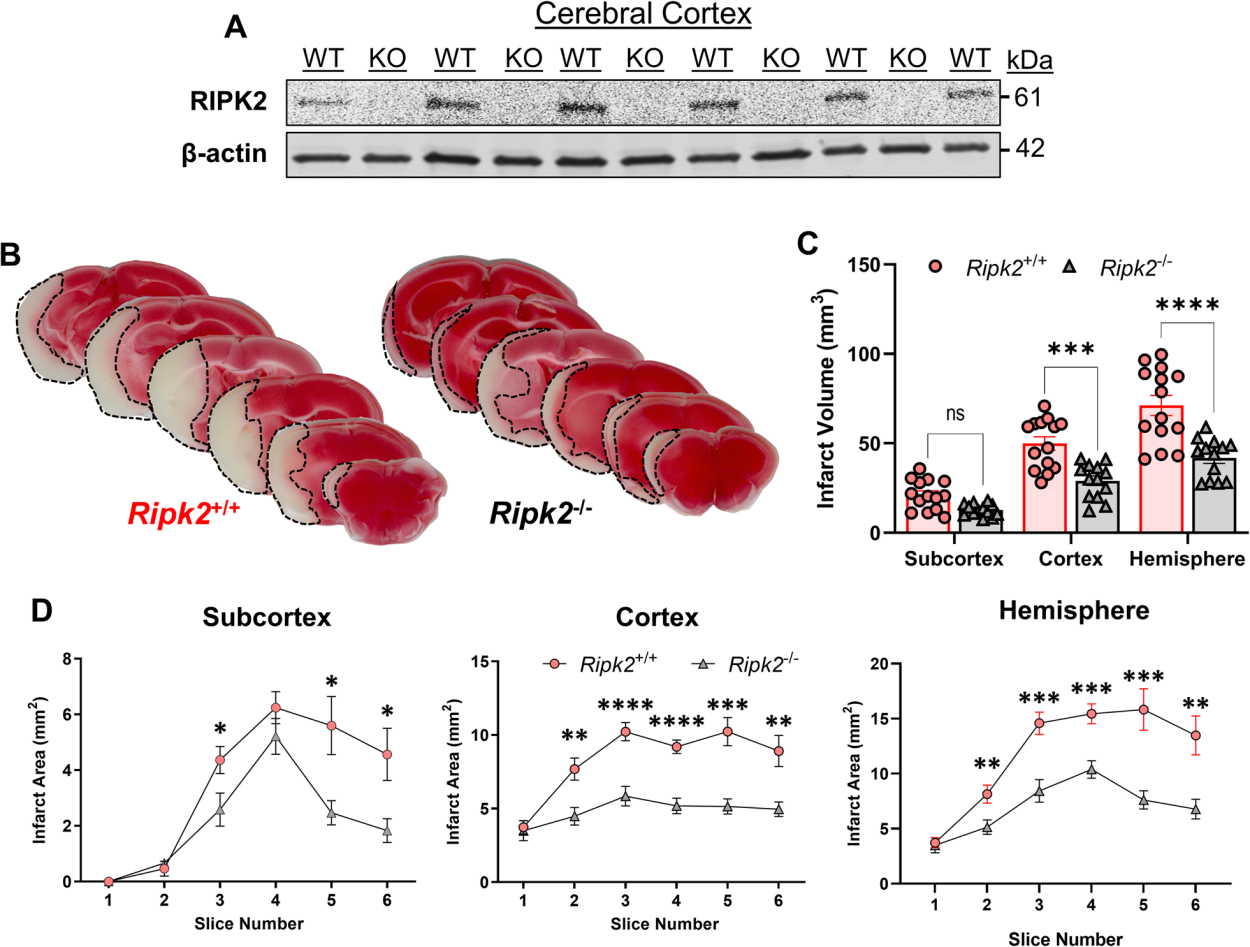
Global deletion of *Ripk2* dramatically reduces infarct volume after transient ischemic stroke. **A** Western blot (WB) of RIPK2 in the cerebral cortex homogenate from *Ripk2*^+/+^ (WT) and *Ripk2*^−/−^ (KO) mice under naïve conditions. **B** Representative TTC staining of 1 mm-thick coronal brain slices from *Ripk2*^+/+^ and *Ripk2*^−/−^ mice 24 h post-tMCAO. **C** Quantitative analysis of total infarction in each brain region. **D** Quantification of the infarction measured from each individual 1-mm-thick slice at the levels of the subcortex, cortex, and total hemisphere. **A** *n* = 5–6/group. **B**–**D** *Ripk2*^+/+^: *n* = 14, *Ripk2*^−/−^: *n* = 13. Statistical differences determined by Student’s *t* test. *P < 0.05, **P < 0.01, ***P < 0.001, ****P < 0.0001

To assess the effect of *Ripk2* deletion on infarct size after stroke, we first induced 45 min-transient middle cerebral artery occlusion (tMCAO) in mice sufficient (*Ripk2*^+/+^) and deficient (*Ripk2*^−/−^) for the *Ripk2* gene and sacrificed the mice 24 h later. The commercially available *Ripk2*^−/−^ mice do not express RIPK2 protein in the cerebral cortex, as detected by Western blot (WB) (Fig. 1A). We found that global deletion of *Ripk2* resulted in a dramatically decreased infarct size 24 h after injury compared to aged-matched *Ripk2*^+/+^ controls (Fig. 1B). Differences in infarct size were observed at the level of the cortex, subcortex, and total hemisphere (Fig. 1C). Major differences were also observed at the level of each individual 1 mm-thick slice in the cortex, subcortex, and total hemisphere (Fig. 1D). The protective effect of *Ripk2* deficiency on infarct size was not due to differences in cerebral vasculature, as we determined there were no differences between the two genotypes in various vasculature parameters using latex blue vessel casting nor in cerebral blood flow measured during tMCAO induction (Additional file 1). These data unveil a critical role for RIPK2 in the progression of acute ischemic stroke brain injury.

### Reduced inflammatory mediators and greater blood–brain barrier preservation in the brains of *Ripk2*^−/−^ mice after ischemic stroke

**Fig. 2:**
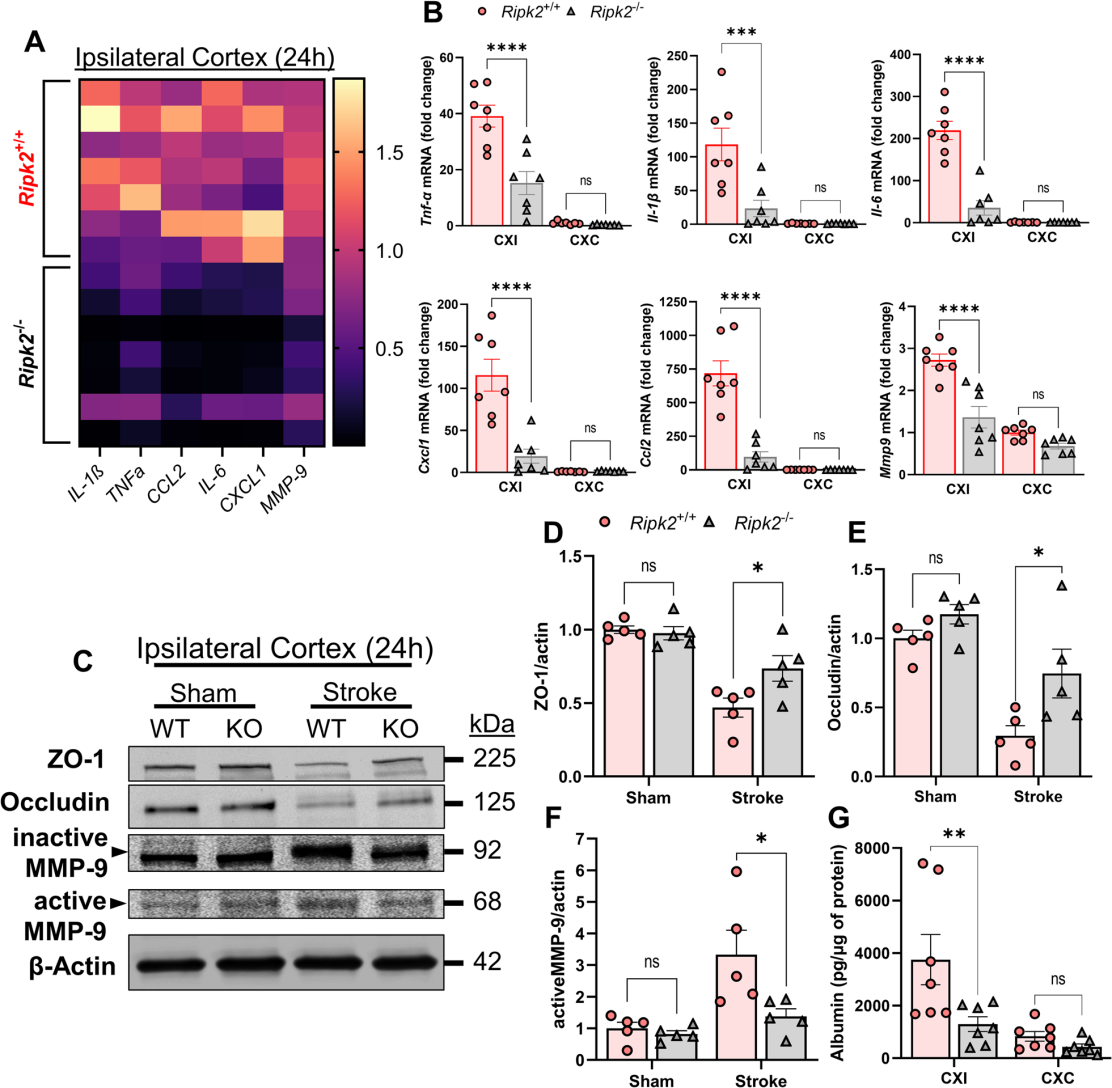
*Ripk2*^−/−^ mice have dramatically lower pro-inflammatory activity and greater BBB preservation in the cerebral cortex 24 h post-stroke compared to *Ripk2*^+/+^ mice. **A** Heat map of inflammation-associated genes in the ipsilateral cerebral cortex comparing the difference in expression between the two genotypes. Horizontal lines denote individual animals, while vertical lines denote genes of interest. **B** Quantification of the heat map in (A), depicting mRNA levels of *Tnfα*, *Il1β*, *Il6*, *Cxcl1*, *Ccl2*, and *Mmp9* in the ipsilateral (CXI) and contralateral (CXC) cortices of the two genotypes 24 h after stroke. **C** Western blot (WB) of the ipsilateral cortex depicting levels of ZO-1, Occludin, and Active-MMP-9, and β-Actin in *Ripk2*^+/+^ (WT) and *Ripk2*^−/−^ (KO) brain homogenate. **D–F** Quantification of WB shown in **C**. **G** ELISA for the detection of the blood serum protein Albumin in the CXI and CXC of WT and KO mice 24 h after stroke, as a measurement of BBB permeability. **A**, **B** *n* = 7/genotype, (**C**–**F**) *n* = 5/genotype, (**G**) *n* = 7/genotype. Statistical differences determined by two-way ANOVA, Turkey’s multiple comparison test. *P < 0.05, ***P < 0.001, ****P < 0.0001

Neuroinflammation plays a critical role in the progression of stroke injury as inflammation contributes to both secondary neuronal damage as well as the recruitment of peripheral immune cells to the site of injury, all of which lead to worse outcomes after stroke [2, 5, 6]. We performed real-time quantitative PCR to detect transcript levels of various inflammatory mediators in the brain between the two genotypes 24 h after ischemic stroke. RT-qPCR of the ipsilateral cerebral cortex (CXI) revealed that *Ripk2*^−/−^ mice have a dramatically lower classical markers of neuroinflammation in the brain after stroke compared to their *Ripk2*^+/+^ counterparts (Fig. 2A, B). In particular, we observed dramatically lower mRNA levels for the classical inflammatory cytokines *Tnfα*, *Il1β*, and *Il6*, chemokines *Cxcl1* and *Ccl2*, and the matrix metalloproteinase *Mmp9* (Fig. 2B). We also found that *Ripk2* knockout mice had greater preservation of tight junction proteins Zona occludens-1 (ZO-1) and Occludin in the ipsilateral cortex 24 h after stroke, which was concurrent with lower levels of active-MMP-9, a major contributor to BBB opening (Fig. 2C–F). This coincided with lower levels of albumin in the ipsilateral cortex of *Ripk2*^−/−^ mice compared to *Ripk2*^+/+^ animals (Fig. 2G). These data indicate greater BBB preservation and a decreased neuroinflammatory signature in *Ripk2* deficient animals in the acute phase of stroke injury compared to WT.

### Reduced early stage immune activation and infiltration in *Ripk2* deficient mice compared to controls

**Fig. 3:**
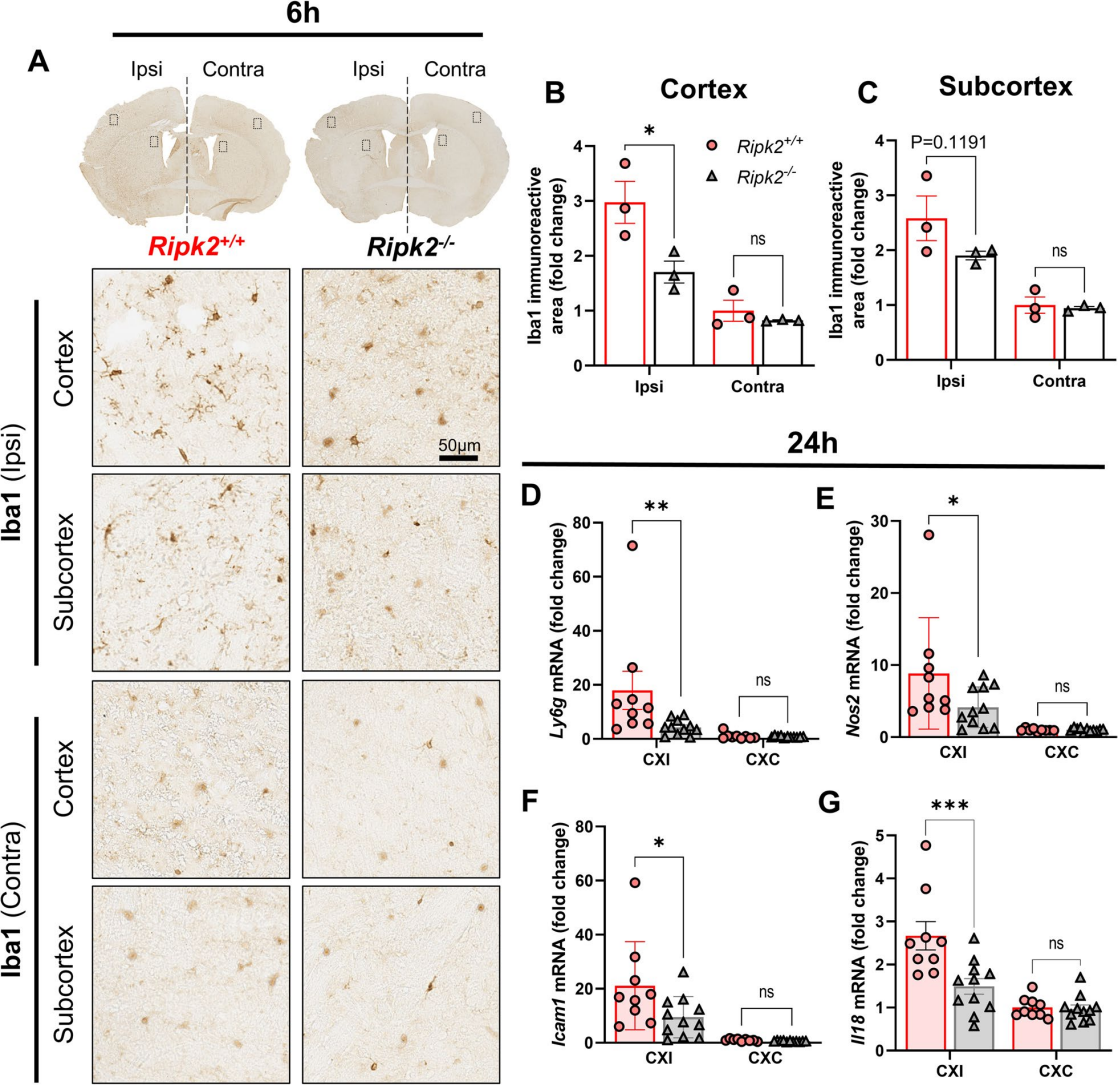
Reduced immune cell infiltration and activation in *Ripk2* deficient mice compared to controls. **A** Iba-1 staining of coronal brain sections 6 h after stroke injury with representative images of the ipsilateral (ipsi) and contralateral (contra) areas of interest. **B, C** Densitometric analysis and quantification of the area of Iba-1 staining in the cortex (**B**) and subcortex (**C**). **D** RT-qPCR for *Nos2*, *Icam1*, and *Ly6g* in the ipsi and contra cortices 24 h after stroke. **A**–**C** *n* = 3/group. D *n* = 9–11/group. Differences determined by two-way ANOVA, multiple comparisons, Turkey’s multiple comparison test. *P < 0.05, ***P < 0.001

We next sought to assess immune cell activation after stroke in the absence of RIPK2. We performed immunohistochemistry for Iba-1, a marker for microglia/macrophages [45], in coronal brain sections of both genotypes collected 6 h after stroke injury. While Iba-1 is not an exclusive microglia marker, examining this marker at 6 h after injury avoids the peak of peripheral macrophage infiltration into the brain. We found that *Ripk2*^−/−^ mice had less ipsilateral Iba-1 positive staining after performing densitometric analysis compared to *Ripk2*^+/+^ controls (Fig. 3A, B). There was also a strong trend toward decreased Iba-1 positivity in the subcortex of *Ripk2*^−/−^ brains compared to controls (Fig. 3C). To further assess immune cell activation and infiltration, we performed additional RT-qPCR for cell markers 24 h after stroke. We found dramatically lower levels of *Ly6g*, a specific marker of neutrophils, in the ipsilateral cortex of *Ripk2*^−/−^ mice compared to *Ripk2*^+/+^ controls (Fig. 3D), as well as lower *Nos2* (Fig. 3E), and *Icam1* (Fig. 3F). These results indicate that RIPK2 plays a role in the microglial response to stroke injury, and its absence reduces markers associated with peripheral immune cell recruitment and activity in the brain after stroke.

### Global deletion of *Ripk2* results in smaller infarct size, favorable behavioral outcomes, and zero mortality 28-day post-stroke

**Fig. 4:**
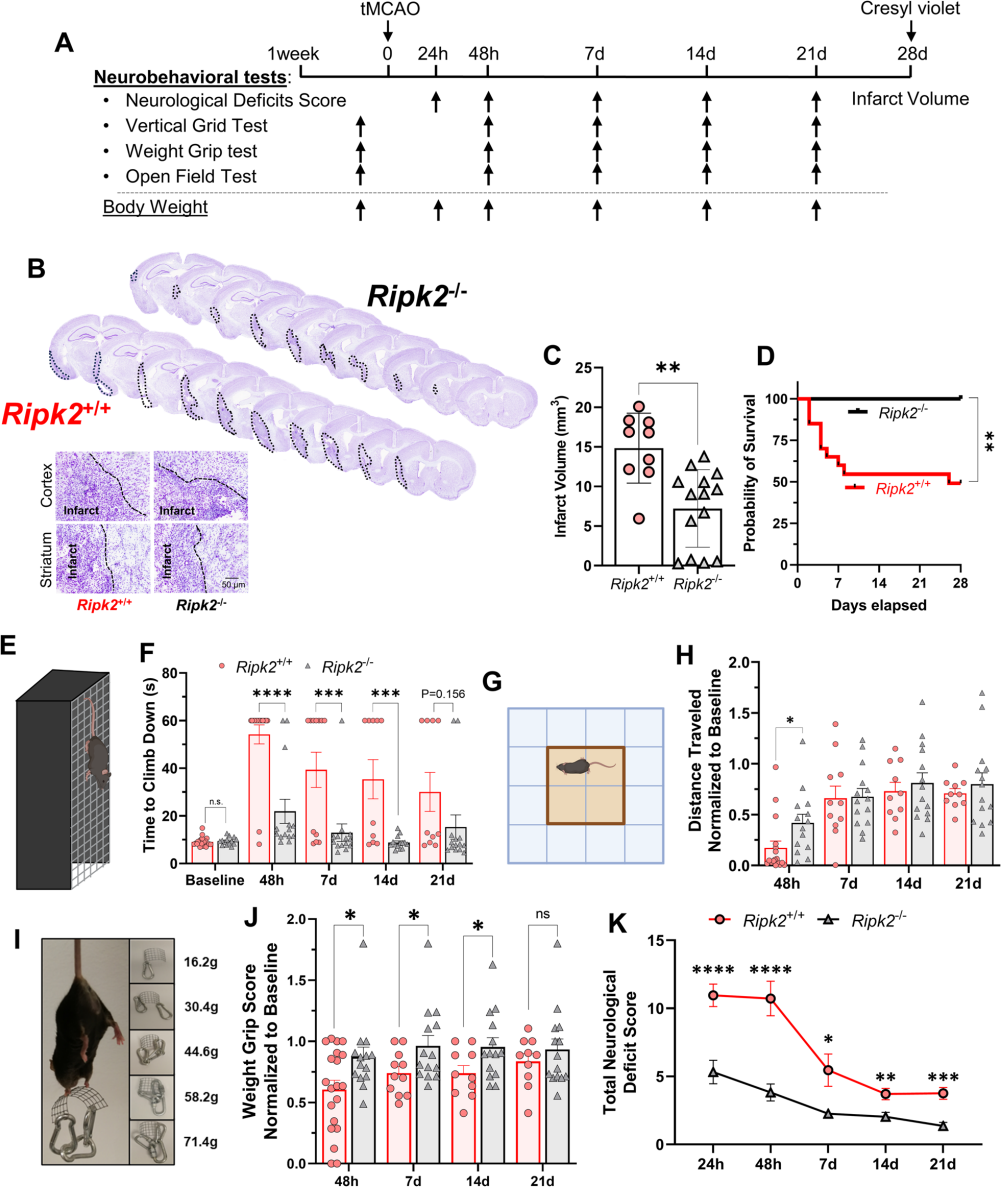
*Ripk2*^−/−^ mice have smaller infarcts, zero mortality, and better behavioral outcomes 28-day post-stroke compared to *Ripk2*.^+/+^ mice. **A** Experimental design for the 28-day, longitudinal study. **B** Cresyl violet staining of 30 μm-thick brain slices at 28 days after stroke with inset depicting delineated areas of the infarct. **C** Quantification of infarct sizes from Cresyl violet-stained sections from each genotype. **D** Kaplan–Meier survival curve out to 28-day post-stroke. **E**, **F** Time required for mice to descend the vertical grid (**E**) after stroke, quantified in **F**. **G,** Distance traveled in the open field chamber (**G**) after stroke, with each value being normalized to that animal’s baseline distance (**H**). **I, J** Weight grip scores determined after stroke. Mice grip weights of increasing mass (**I**) and scores were quantified (**J**). **K** Total neurological deficit score assigned at indicated timepoints after stroke. **B**, **C** *n* = 9–14/genotype. **F**–**K** *n* = 14–21/genotype at 24 h and 48 h, *n* = 11–14/genotype at 7d, *n* = 10–14/genotype at 14d and 21d. **C** Student’s *t* test. **D** Gehan–Breslow–Wilcoxon test. **F**, **H** Two-way ANOVA. **J**, **K** Mann–Whitney test. *P < 0.05, **P < 0.01, ***P < 0.001, ****P < 0.0001

We next sought to determine the long-term effects of *Ripk2* deletion in animals after stroke. We performed a longitudinal study of WT and *Ripk2*^−/−^ mice subjected to 45 min-tMCAO. At various timepoints after stroke, we subjected these animals to behavioral testing (Fig. 4A). We performed Cresyl violet staining of serial brain sections from animals following termination of the study at 28 days and calculated infarct volumes by subtracting the healthy area on the ipsilateral side from the contralateral hemisphere. In this way, we accounted for the possibility of smaller infarct volumes resulting from enhanced clearance of necrotic tissue. We found that *Ripk2*^−/−^ mice have dramatically reduced infarct size compared to their WT counterparts (Fig. 4B, C). Surprisingly, we found that global deletion of *Ripk2* resulted in zero mortality out to 28-day post-stroke, whereas WT animals suffered 50% mortality (Fig. 4D). *Ripk2*^−/−^ mice displayed a greater ability to descend the vertical grid (Fig. 4E) at nearly all testing timepoints after stroke (Fig. 4F). *Ripk2*^−/−^ mice initially travel a greater distance in the open field (Fig. 4H) and show no difference in time spent in the center of the chamber (Additional file 3). Using the weight grip test (Fig. 4I), we found that *Ripk2*^−/−^ animals also displayed greater preservation of grip strength after stroke, as evidenced by their improved grip scores (Fig. 4J). Finally, *Ripk2*^−/−^ mice scored markedly lower on the neurological deficits score (NDS) test compared to WT mice (Fig. 4K), indicating greater preservation of their neurological functioning and motor coordination after stroke. Neurological deficit scores in *Ripk2*^+/+^ and *Ripk2*^−/−^ mice at different timepoints during the 28-day longitudinal study are found in Additional file 2.

### *Ripk2* deficiency protects male and female aged animals from acute stroke injury

**Fig. 5:**
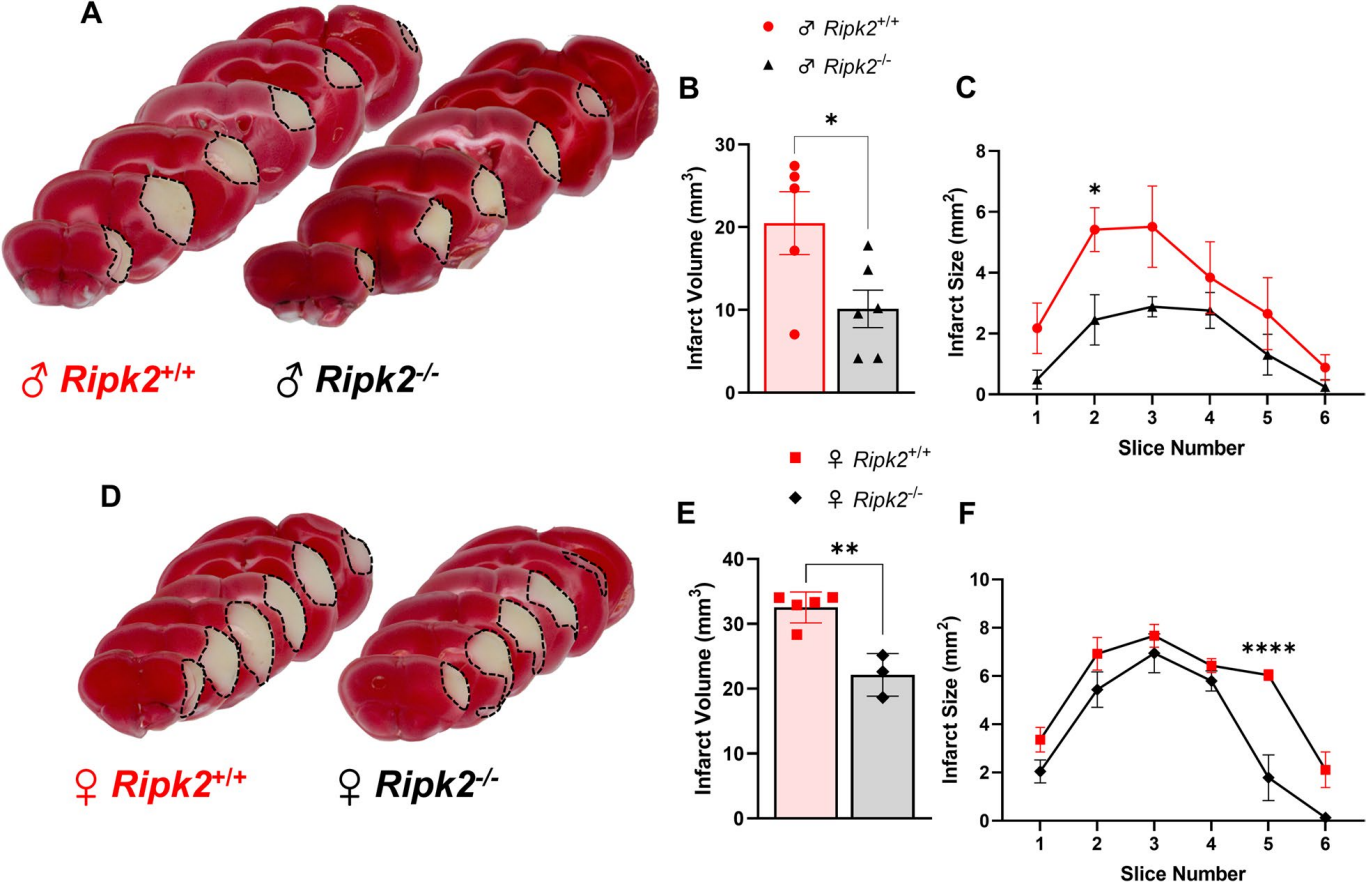
*Ripk2* deficiency protects male and female mice from stroke injury 48 h after permanent MCAO. **A** TTC staining of brains of aged (18–24 months) male *Ripk2*^+/+^ and *Ripk2*^−/−^ mice 48 h after stroke. **B** Quantification of infarct size from TTC staining in **A**. **C** Infarct size quantified for each coronal section. **D** TTC staining of aged female *Ripk2*^+/+^ and *Ripk2*.^−/−^ mice 48 h after stroke. **E** Quantification of infarct size from TTC staining in **D**. **F** Infarct size quantified for each coronal section. **A**–**C** *n* = 5–6/genotype. **D**–**F** *n* = 3–5/genotype. Student’s *t* test. *P < 0.05, **P < 0.01, ****P < 0.0001

To add greater translational relevance to the protective role of *Ripk2* deletion in stroke, we wanted to know how these animals would perform with both the comorbidity of age and a permanent occlusion model of stroke. To do so, we subjected both aged male and female mice to permanent middle cerebral artery occlusion (pMCAO) and assessed the effect of stroke on infarct size during the acute stage of injury. We found that aged male *Ripk2*^−/−^ mice subjected to pMCAO displayed less brain injury at 48 h after stroke as detected by TTC staining compared to aged-matched WT controls (Fig. 5A–C). We also found that this protective effect extended to aged female *Ripk2*^−/−^ mice who also showed less brain injury 48 h after pMCAO compared to aged-matched WT controls (Fig. 5D–F). This data indicates that the protective effect of *Ripk2* deletion is not restricted to models of stroke that allow for recanalization, such as the tMCAO model, but extends to permanent occlusion models of stroke. This data also reveals a protective effect of *Ripk2* global deletion in aged animals of both sexes.

### Iba1 positive cells express RIPK2 after stroke and microglia derived from *Ripk2*^−/−^ animals elicit differential gene expression 24 h following ischemic stroke

**Fig. 6:**
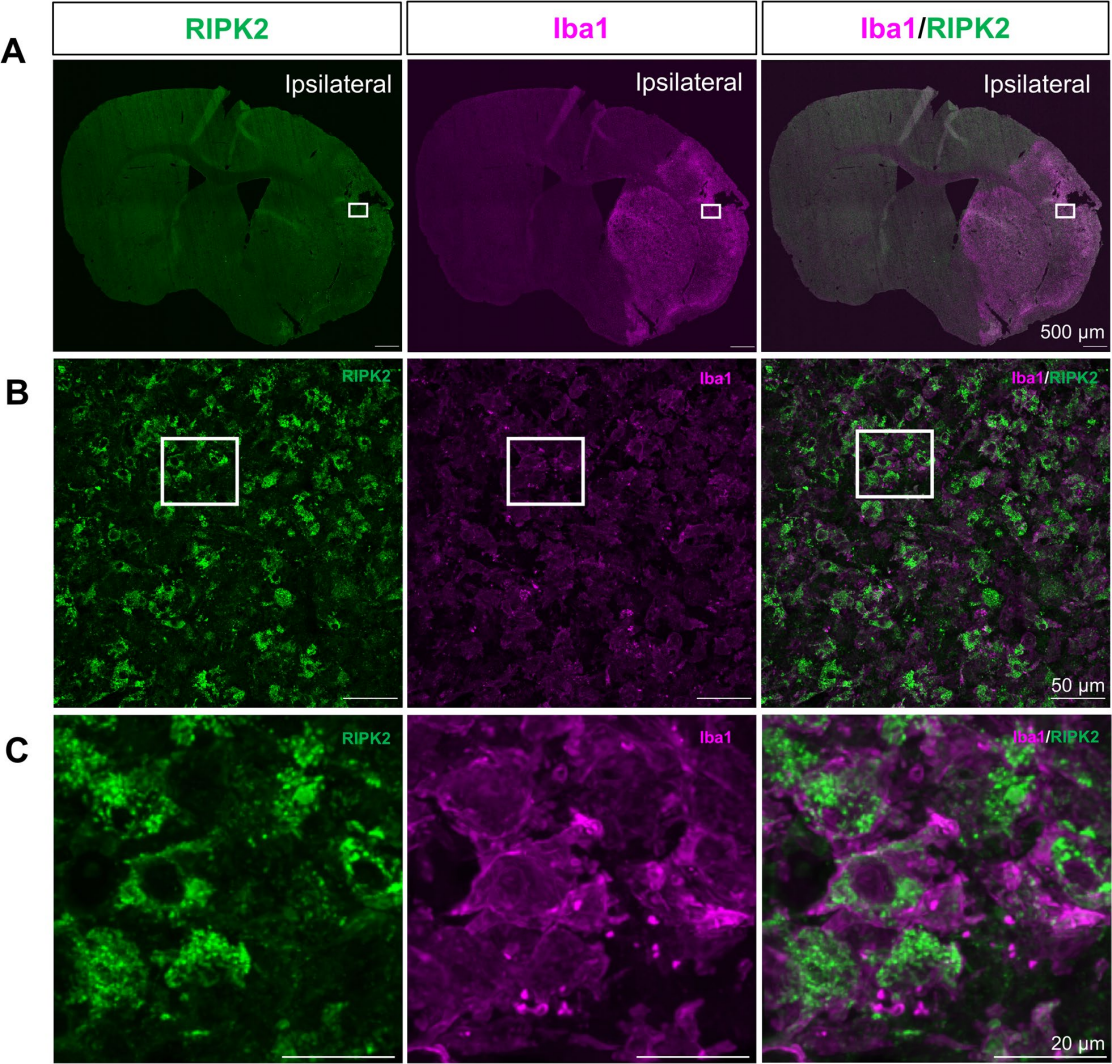
RIPK2 is highly co-expressed with Iba1 positive cells in the ipsilateral cortex after stroke. **A** Representative coronal section images of RIPK2 and Iba1 staining in the brain 7 day post-stroke. Open squares indicate the area for micrographic examination in **B**. **B** Cortical region of interest showing a high degree of overlapping signal between RIPK2 and Iba1, with open squares denoting the area examined at higher magnification in **C,** where Iba1 positive cells show distinct colocalization with RIPK2. Images representative of *n* = 3

Microglia play an important role in the early progression of stroke injury as they are among the first responders to the damage that occurs immediately following the cessation of blood flow to the brain. Their initial response sets the tone for the proceeding inflammatory response. We determined that RIPK2 is highly expressed in Iba1-positive cells in the ipsilateral cortex after stroke (Fig. 6). Iba1 is a marker for microglia/macrophages [45] and is upregulated by macrophages during their activation [46].

**Fig. 7:**
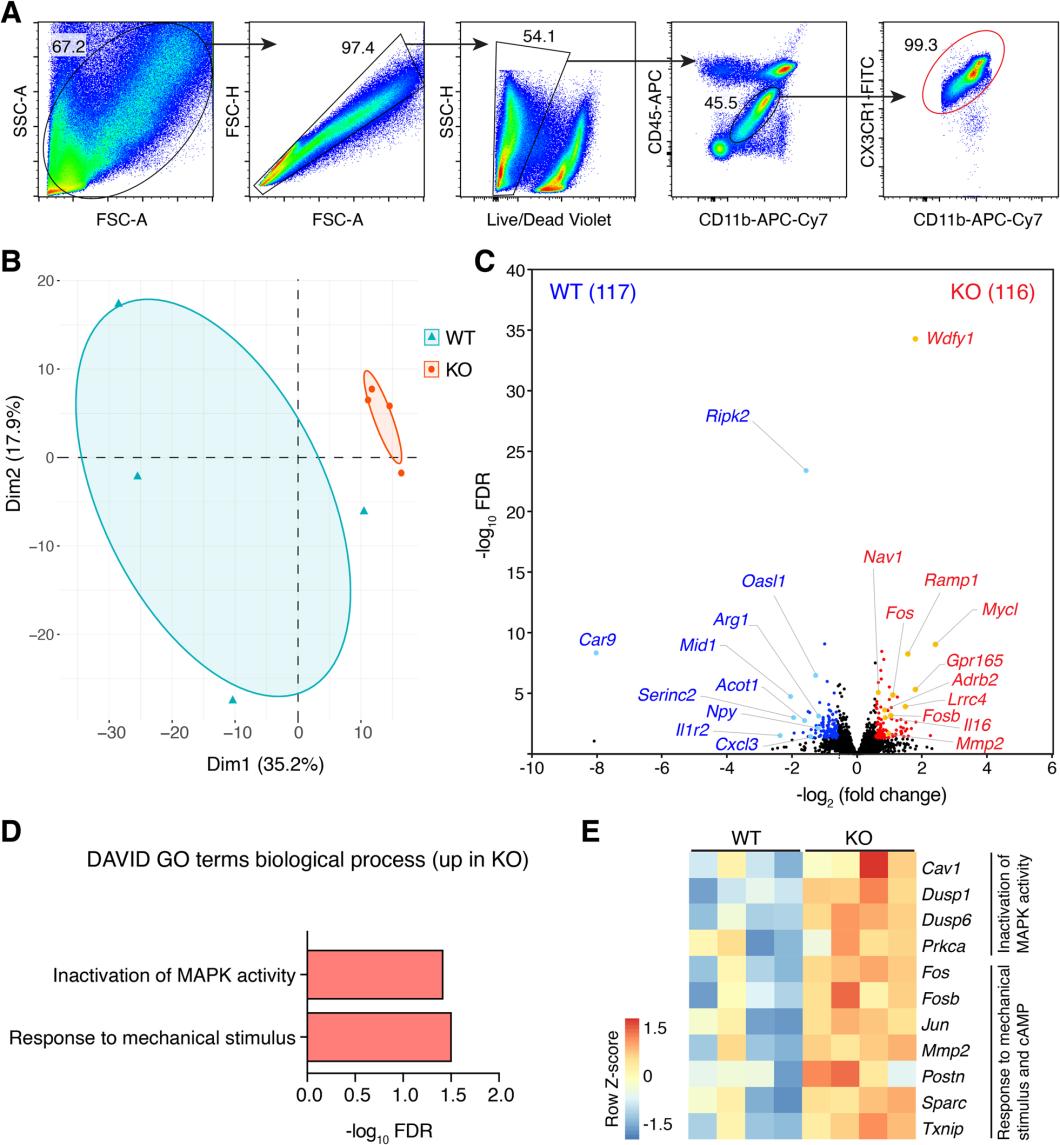
RNA-sequencing confirms the role of *Ripk2*-deficient microglia in ischemic stroke injury.** A** Flow cytometric gating strategy for microglia. **B** Principal component analysis (PCA) plot of transcriptomes of microglia isolated from WT and *Ripk2* KO mice following ischemic stroke. **C** Volcano plot of differentially expressed genes (DEGs, fold change > 1.5, FDR < 0.05) between WT and KO mice. The number of genes enriched in the indicated groups is shown in parentheses. **D** DAVID Gene Ontology (GO) analysis for biological process using upregulated DEGs in KO group. No GO terms (FDR < 0.1) were found when using enriched DEGs in the WT group. **E** Heat map of DEGs related to pathways shown in **C**. *n* = 4/genotype. To correct for differences in infarct volume, animals were subjected to different times of occlusion (45 min for WT, 60 min for *Ripk2* KO). Microglia were isolated 24 h post-stroke

As such, we wanted to assess the transcriptional response of microglia derived from *Ripk2* globally deficient mice after stroke. We controlled for observed differences in infarct sizes between genotypes by subjecting the mice to differing times of tMCAO (Additional file 4). Data demonstrated that microglia isolated from *Ripk2* KO mice displayed a unique global transcriptome as compared to the WT controls (Fig. 7B), resulting in a total of 233 differentially expressed genes (DEGs) (Fig. 7B, C). The top DEGs enriched in KO mice included neuronal activity-associated transcripts (e.g., *Nav1*, *Ramp1* and *Adrb2*) and AP-1 transcription factors (*Fos*, *Fosb* and *Jun*), whereas the expression of inflammatory molecules (e.g., *Npy* and *Cxcl3*) was reduced (Fig. 7C). Gene set enrichment analysis further revealed elevated pathways and genes associated with inactivation of MAPK activity in the KO microglia (Fig. 7D, E), highlighting a negative role of RIPK2 in controlling ischemic stroke-induced neuroinflammation. This data prompted us to further investigate the extent to which microglia contribute to stroke injury through RIPK2 utilization.

### Selective deletion of *Ripk2* from microglia reduces infarct size and protects against stroke-induced BBB damage

**Fig. 8:**
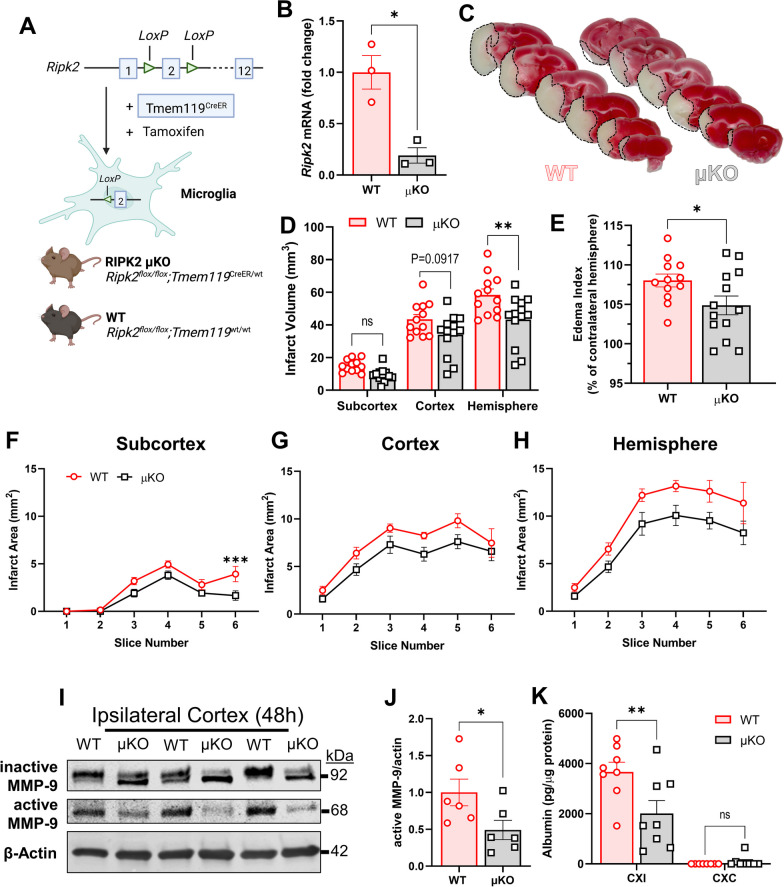
Selective deletion of *Ripk2* from microglia reduces infarct size and preserves BBB 48 h after stroke. **A** Schematic paradigm of microglial-specific *Ripk2* conditional knockout (μKO) animal generation. **B** Quantification of *Ripk2* expression in WT- and μKO-derived microglia. **C** TTC staining of WT and μKO brain slices 48 h after tMCAO. **D** Quantification of total infarct sizes at the subcortical, cortical, and hemisphere levels. **E** Edema index represented as the ipsilateral hemisphere percentage of the contralateral hemisphere. **F–H** Quantification of the infarct of each individual 1 mm-thick slice in the subcortex (**F**), cortex (**G**), and hemisphere (**H**). **I** Representative WB of active and inactive MMP-9 in the ipsilateral cortex of WT and μKO 48 h after stroke.** J** Quantification of WB in **I**. **K** Albumin levels in the cerebral ipsilateral (CXI) and contralateral (CXC) cortices of WT and μKO mice 48 h after stroke. **B** *n* = 3/group, *Ripk2* mRNA expression normalized to housekeepers *18s* and *Cyc1*. **C**–**H** *n* = 13/group. **I**, **J** *n* = 6/group. **K** *n* = 8/group. **B**, **E**, **J** Student’s *t* test. **D**–**H**, **K** Two-way ANOVA. *P < 0.05, **P < 0.01

To date, there have been no reports that directly address the specific role of RIPK2 in microglia due to the lack of genetic models targeting these cells. We created a paradigm, where microglia are rendered genetically incapable of propagating RIPK2 signaling to explore the RIPK2–microglial contribution to stroke injury. We generated mice, where *Ripk2* is selectively deleted in microglia under the regulation of the *Tmem119* promoter using the Cre-loxP system (Fig. 8A). After confirming the *Ripk2*^flox/flox^;*Tmem119*^CreERT2/wt^ (μKO) mice displayed sufficient deletion of *Ripk2* in isolated microglia following tamoxifen injection (Fig. 8B), we subjected μKO and appropriate control mice (WT) to 45 min-tMCAO and found a significant reduction in total hemisphere infarct size 48 h after stroke in our μKO mice compared to WT (Fig. 8C, D). There was also a significant decrease in the edema index between WT and μKO mice after stroke, as represented by the ipsilateral percentage of the contralateral area (Fig. 8E). There were no significant differences in cerebral blood flow (CBF) at baseline, during occlusion, or after reperfusion between WT and μKO genotypes (Additional file 5). We further characterized stroke injury at the subcortical, cortical, and total hemisphere levels and calculated the infarct for each coronal brain slice (Fig. 8F–H). Western blotting revealed decreased levels of active MMP-9 in the CXI of μKO mice compared to WT 48 h after stroke (Fig. 8I, J). To assess BBB permeability, we employed an ELISA to detect the presence of the blood-borne protein albumin in the cerebral cortex. We compared the levels detected in the ipsilateral and contralateral cortices of the two genotypes. We found a markedly decreased albumin level in the CXI of the μKO mice compared to the WT (Fig. 8K), indicating greater BBB preservation after stroke. Examined in total, this data reveals a neuroprotective effect of microglial–*Ripk2* deficiency.

### Microglial deletion of *Ripk2* improves acute behavioral outcomes after stroke

**Fig. 9:**
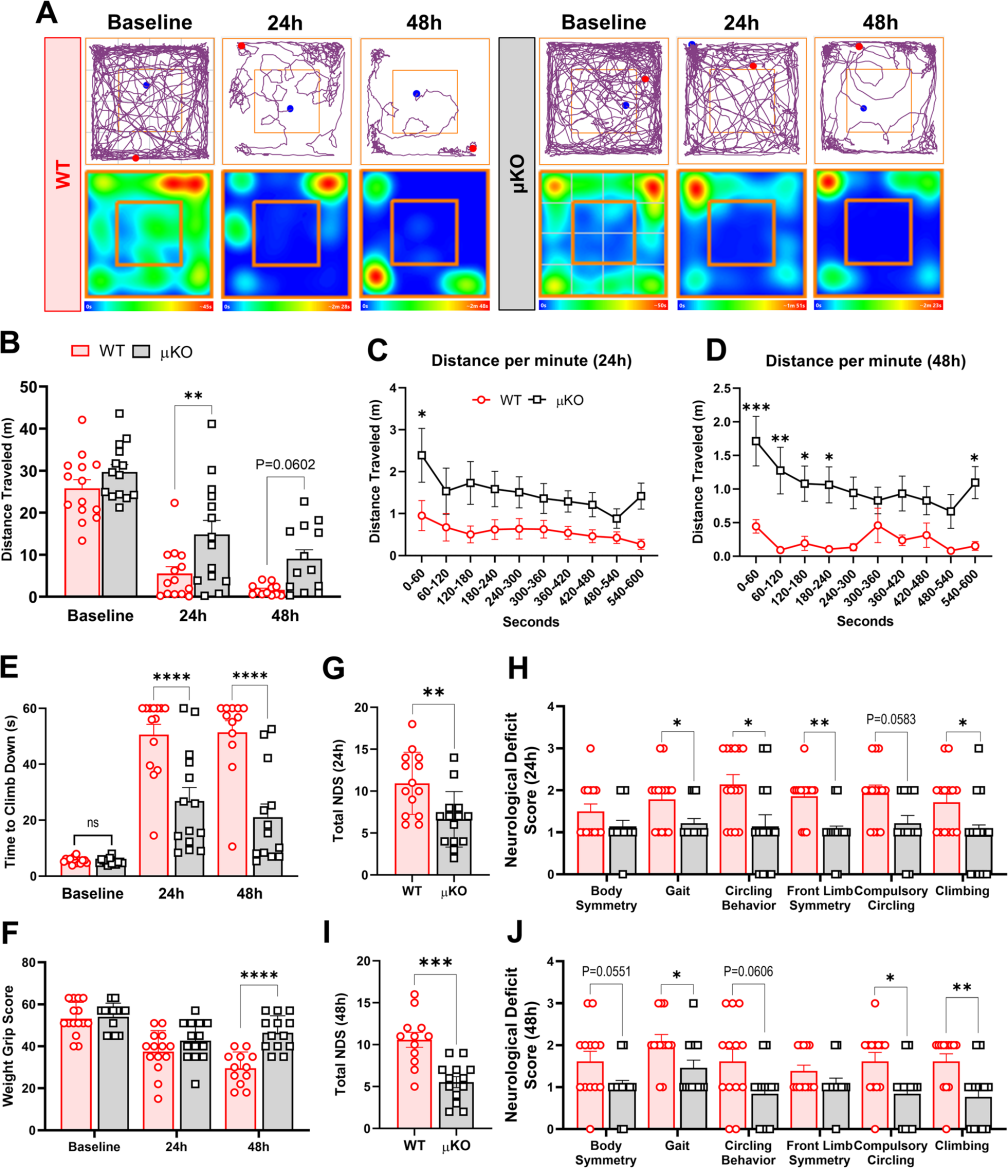
Microglial deletion of *Ripk2* improves acute behavioral outcomes after stroke. **A–D** Locomotor function was assessed by the open field test. Graphical depictions of track plots and corresponding heatmaps (**A**) reveal greater distances traveled by *Ripk2* conditional knockout mice (μKO) compared to wild type (WT) at 24 h and 48 h after stroke (**B**), with clear separation between groups when examining each minute of the open field test (**C**, **D**). **E** Weight grip scores. **F** Time required to descend the vertical grid. **G–J** Neurological deficits scores (NDS). Total score at 24 h (**G**) with scores of individual parameters in **H**. Total score at 48 h (**I**) with individual scores in **J**. **A**–**J** *n* = 14–15/group. **A**–**E** Two-way ANOVA (**G**–**J**) Mann–Whitney test. *P < 0.05, **P < 0.01, ***P < 0.001 ****P < 0.0001

To determine the effects of microglial *Ripk2* deletion on post-stroke behavioral outcomes, we subjected μKO mice and WT controls to a battery of behavioral tests during the acute 48 h phase of injury. We found that the μKO mice had enhanced capability to travel further distances in the open field test compared to the WT at 24 h and showed a strong trend toward significance at 48 h after injury (Fig. 9A–D). The μKO animals displayed a greater ability to descend the vertical grid (Fig. 9E), indicating greater preservation of both their strength and motor coordination after stroke compared to the WT. μKO also showed greater preservation of grip strength as they performed better on the weight grip test at 48 h post-stroke compared to WT mice (Fig. 9F). Finally, we observed that the μKO mice scored significantly better on the NDS assessment than WT mice at both 24 h and 48 h after stroke (Fig. 9G–J). No differences in mortality rates were found between WT and μKO mice (Additional file 5). Behaviorally, μKO mice retained better motor function when assessed across the various behavioral paradigms compared to WT mice during the acute 48 h period of stroke injury, implicating that microglia play a specific role in propagating stroke-induced behavioral deficits through RIPK2.

### Reduction in microglial *Ripk2* expression reduced neuroinflammatory gene transcription after ischemic stroke

**Fig. 10:**
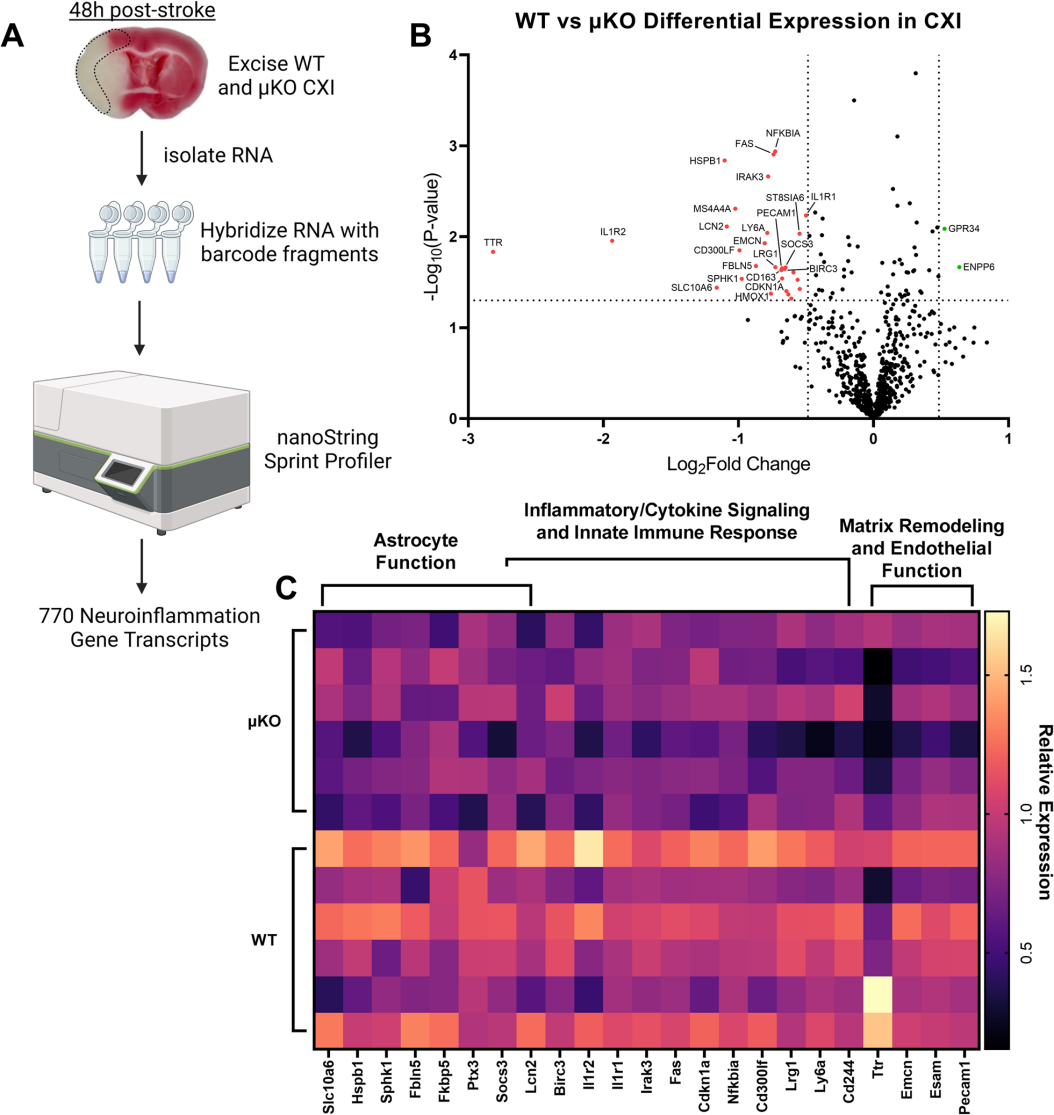
Reduction in Microglial *Ripk2* expression reduces neuroinflammatory gene expression after ischemic stroke. **A** Schematic of our isolation of RNA from the ipsilateral cortex (CXI) of WT and μKO mice and nanoString protocol. **B** Volcano plot displaying gene expression levels in the CXI 48 h after stroke. *P* values were – Log_10_ transformed; statistically significant genes fall above the horizontal line. Highly differentially expressed genes (with fold change >  ± 1.4) fall to either side of the vertical lines on either side of the zero on the *x*-axis. Relevant genes are labeled. **C** Heat map of relevant differentially expressed genes with pathway analysis. Columns represent an individual sample from each genotype

We isolated total RNA from the ipsilateral cortex of WT and μKO 48 h after stroke and subjected it to the commercially available nanoString^®^ nCounter^®^ Neuroinflammation Panel to assess neuroinflammatory gene transcription at the site of stroke injury between our two genotypes (Fig. 10A). Differential gene expression analysis revealed a total of 31 differentially expressed genes (29 downregulated, 2 upregulated) with fold change of ± 1.4 and a *P* value of < 0.05 (Fig. 10B). Performing pathway analysis, we observed a marked decrease in genes associated with reactive astrocytic function in our μKO mice compared to WT as well as reductions in inflammatory/cytokine signaling and innate immune response genes as well as genes associated with matrix remodeling and endothelial function (Fig. 10C). These data provide critical insights into the mechanisms by which the absence of *Ripk2* from microglia improves animal outcomes after stroke.

## Discussion

In this study, we utilized genetic approaches to define the role of RIPK2 in ischemic stroke. Our findings provide compelling mechanistic evidence that RIPK2 plays a key role in promoting the neuroinflammatory response to ischemic brain injury. Our initial evidence highlighted the dramatic decrease in infarct size after stroke in mice, where *Ripk2* is globally absent. As neuroinflammatory processes contribute to the expansion of stroke injury in the acute phase, we next determined that the *Ripk2* KO mice displayed robust decreases in neuroinflammatory gene transcription concurrent with indicators of greater BBB preservation compared to their WT counterparts. *Ripk2*^−/−^ mice had decreased levels of Iba1^+^ cells in the ipsilateral cortex 6 h after stroke compared to the *Ripk2*^+/+^ mice, implicating a diminished microglia response to injury. We also observed a significant number of RIPK2^+^, Iba1^+^ double-positive cells in the ipsilateral cortex after stroke. These data prompted us to ask whether microglia utilize RIPK2 to promote neuroinflammatory processes in ischemic stroke. As such, we next generated mice deficient for *Ripk2* in microglia using the Cre-lox system under the control of the *Tmem119* promoter and subjected them to stroke.

Importantly, we conducted a longitudinal study and showed that *Ripk2*^−/−^ mice have reduced infarct volumes and zero mortality out to 28-day post-stroke compared to *Ripk2*^+/+^ mice. *Ripk2*^−/−^ mice also performed better on behavioral tests, especially the vertical grid and weight grip tests, and received significantly better neurological deficit scores. Grip strength, as assessed by both the vertical grid and weight grip tests, is a critical metric to post-stroke recovery and is associated with longevity and survival in old age [47–49]. Post-stroke cognitive impairment is a common post-stroke outcome [50], and interventions that reduce post-stroke neurological deficits, such as what we observed with *Ripk2* global deletion, are greatly needed. Compared to C57BL/6J mice, we observed above-average mortality in our *Ripk2*^+/+^ cohort (50%, 10/20 total) which may be due to the use of *Ripk2*^+/+^ littermate controls, emphasizing the importance of proper controls in experiments with genetic strains of animals. We also showed that the absence of *Ripk2* elicits neuroprotective effects after stroke regardless of the age or sex of the animals. Our future studies will further explore the effects of *Ripk2* deletion in aged animals of both sexes.

As the brain's predominant innate immune cell, microglia play a critical role in the initial inflammatory response to stroke injury. In our μKO studies, we showed that microglia utilize RIPK2 to promote stroke injury and that deleting the *Ripk2* gene specifically in microglia results in decreased infarct size and reduced markers of BBB disruption. Of most importance, μKO mice displayed dramatically improved behavioral outcomes in the acute phase of stroke injury. While the protective effect observed in our μKO mice was not as strong as the effect observed in the global KO animals, the fact that we observed an effect on infarct size and especially behavioral outcomes strongly implicates RIPK2 in the initial microglia response to ischemia-induced injury. We speculate that there may be a greater role for RIPK2 utilization by peripheral immune cells, who are known to express RIPK2 [26, 51, 52], in promoting stroke injury, and that this may explain the more powerful protective effect observed in our *Ripk2*^−/−^ compared to our μKO animals. One limitation of our μKO study and *Ripk2*^−/−^ longitudinal study is that only male mice were included in the analysis. Further studies will assess the long-term behavioral outcomes in our μKO mice after stroke, as well as assess the biological variables of sex and age.

An accumulation of recent evidence associates gut permeability and the leakage of highly immunogenic bacteria and their products from the gut into the systemic circulation after ischemic stroke [8–15]. As RIPK2 is known to play an important role in the signaling of NOD1/2 intracellular PRRs, which respond to bacterial products [16], we speculate that peripheral immune cells may be primed toward a more pro-inflammatory phenotype during the sequelae of stroke and may exert hyper-inflammatory activity upon their infiltration into the brain. We will further investigate the contribution of peripheral immune cell usage of RIPK2 by inducing stroke in animals, where *Ripk2* is specifically deleted from myeloid-derived immune cells.

Pharmacological inhibition or degradation of RIPK2 in vivo may also prove beneficial in improving stroke outcomes. Indeed, RIPK2 is an attractive drug target for many inflammatory conditions, particularly for inflammatory conditions of the gut, such as Crohn’s disease and inflammatory bowel disease, as well as early onset sarcoidosis, Blau syndrome, inflammatory breast cancer, and multiple sclerosis [53–55]. Drug discovery efforts continue to expand the arsenal of RIPK2-inhibiting compounds focused on treating these inflammatory conditions [56–61]. Due to its long half-life [62], RIPK2 is also a target of interest for proteolysis-targeting chimera (PROTAC)-mediated degradation, such that there has been significant development of compounds allowing for selective pharmacological degradation of RIPK2 in vivo [63, 64]. Repurposing currently available RIPK2 inhibitors/degraders for post-stroke therapy offers an exciting novel strategy for targeted treatment.

RNA-sequencing analysis revealed important differences between microglia derived from our *Ripk2*^−/−^ mice and *Ripk2*^+/+^ mice. Of note, microglia from *Ripk2*^−/−^ mice showed marked increases in genes associated with the inactivation of MAPK activity, which may be due to RIPK2’s ability to phosphorylate and activate TAK1 [22], thereby increasing MAPK pathway activity in *Ripk2*^+/+^ microglia relative to *Ripk2*^−/−^ microglia. Interestingly, *Cav1*, a well-documented anti-inflammatory modulator encoding for the protein Caveolin-1 [65–67], was highly upregulated in *Ripk2*^−/−^ microglia. Caveolin-1 has been associated with the inhibition of MMP-9 and subsequent preservation of the BBB in ischemic rodent models [68, 69]. *Ripk2*^−/−^ microglia also displayed increases in genes associated with the response to mechanical stimulus.

Analysis of 770 neuroinflammation-associated genes in the ipsilesional cortex of our μKO and WT control mice 48 h after stroke revealed insights into potential mechanisms of protection in mice, where *Ripk2* is specifically deleted from microglia. Pathway analysis revealed decreases in genes related to astrocyte function, especially decreases in *Lcn2*, which has many neuroinflammatory effects in the context of stroke [70, 71], in the μKO compared to WT. Genes associated with inflammatory cytokine signaling and innate immune response were also significantly downregulated in the μKO CXI, particularly *Il1r1*, *Il1r2*, and *Nfkbia*, indicating that the absence of *Ripk2* in microglia has profound effects on the immune response to stroke injury, further strengthening our claim that the detrimental effect of RIPK2 is mediated by promoting neuroinflammation after stroke.

## Conclusions

This study assessed the acute and long-term behavioral outcomes of *Ripk2*^−/−^ mice after experimental ischemic stroke and examined the extent to which microglia contribute to ischemic stroke injury by utilizing RIPK2. We provide new evidence that RIPK2 plays a pathological role in the progression of stroke injury by promoting neuroinflammation and increasing infarct size in both the acute and late stages of injury. This study is significant, because it identifies RIPK2 as a novel therapeutic target for the treatment of ischemic stroke and other neuroinflammatory conditions. We hope that our generation of *Ripk2*^flox/flox^ mice will spur further investigation into the various roles RIPK2 plays in different cell types and disease contexts. Our future experiments will explore whether pharmacological inhibition of RIPK2 can recapitulate the neuroprotective effects observed in this study.

### Supplementary Information

**Additional file 1: ***Ripk2*^−^/^−^ mice show no differences in cerebral vessel parameters at baseline or cerebral blood flow during stroke surgery. **A **Representative vessel casting images depicting the superior (top) and inferior (bottom) views of *Ripk2*^−^/^−^ and *Ripk2*^+/+^ following latex vessel casting. Square inserts depict method for measuring the distance from baseline and for counting the number of anastomoses. **B **The number of anastomotic connections between vessels originating from the brain midline to the branches of the middle cerebral artery. **C **The distance from anastomotic connections to the brain midline. **D **The diameter of various cerebral arteries. **E **Cerebral blood flow measured during tMCAO surgery for both genotypes. (A–D) n = 4–5/group. Differences determined by Student’s *t* test (B) or two-way ANOVA (C, D). Abbreviations: ACA: anterior cerebral artery, MCA: middle cerebral artery, ICA: internal carotid artery, PCA: posterior cerebral artery.

**Additional file 2: **Differences in neurological deficit score parameters between *Ripk2*-/- and *Ripk2*^+/+^ mice during the 28-day longitudinal study. **A–E **Neurological deficit scores for each of the five parameters, with total scores for each indicated timepoint. Scores were recorded at 24 h (A), 48 h (B), 7d (C), 14d (D), and 21d (E) post-stroke. n = 14–21 per group at 24 h and 48 h, n = 11–14/genotype at 7d, n = 10–14/genotype at 14d and 21d. Differences determined by Mann–Whitney test. * P < 0.05, ** P < 0.01 *** P < 0.001 **** P < 0.0001

**Additional file 3: **No differences in the time spent in the center of the open field chamber between genotypes. Recordings for the time mice spent in the center of the open field chamber were taken at baseline then at 48 h, 7-day, 14-day, and 21-day post-stroke. n = 10–14mice/group. No differences were determined via two-way ANOVA, multiple comparisons.

**Additional file 4: ***Ripk2*^−^/^−^ mice experience similar infarct volumes with 60min of occlusion compared to *Ripk2*^+/+^ with 45min of occlusion. To correct for differences in infarct volume before performing our bulk RNAseq on microglia, the *Ripk2*^−^/^−^ mice were subjected to an additional 15 min of occlusion time compared to *Ripk2*^+/+^ mice. This produced a similar level of infarction between the two genotypes. n = 5–10mice/group. No differences determined by Student’s *t* test.

**Additional file 5 A: **No observed differences in cerebral blood flow during the course of tMCAO surgery in WT vs μKO mice. **B **No statistical differences in survival during the 48h period following stroke induction; however, μKO mice suffered 1 animal loss compared to 3 in the WT group. n=13/genotype.

**Additional file 6:** Unedited Western blots.

## Data Availability

All the raw data supporting the conclusions of the paper have been deposited at Mendeley Data. A link to our data set can be found here: https://data.mendeley.com/datasets/72dkxdrjmw/1.

## Abbreviations

ACA: Anterior cerebral artery
BBB: Blood–brain barrier
CARD: Caspase activation and recruitment domain
CBF: Cerebral blood flow
CCA: Common carotid artery
CXI: Ipsilateral cerebral cortex
DAMP: Damage-associated molecular pattern
DEG: Differentially expressed gene
ECA: External carotid artery
FACS: Fluorescence-activated cell sorting
FDR: False discovery rate
GO: Gene ontology
ICA: Internal carotid artery
IL: Interleukin
MAPK: Mitogen-activated protein kinase
MCAO: Middle cerebral artery occlusion
μKO: Microglia-specific Ripk2 conditional knockout
NF-κB: Nuclear factor-kappa B
NOD: Nucleotide-binding oligomerization domain
PAMPs: Pathogen-associated molecular patterns
PRRs: Pattern recognition receptors
RIPK2: Receptor-interacting protein kinase 2
TAK1: Transforming growth factor beta-activated kinase 1
TTC: 2,3,5-Triphenyltetrazolium chloride
WT: Wild type
ZO-1: Zona occludens-1

## References

[1] Collaborators GBDS (2021). Global, regional, and national burden of stroke and its risk factors, 1990–2019: a systematic analysis for the Global Burden of Disease Study 2019. Lancet Neurol.

[2] Yang C, Hawkins KE, Dore S, Candelario-Jalil E (2019). Neuroinflammatory mechanisms of blood–brain barrier damage in ischemic stroke. Am J Physiol Cell Physiol.

[3] Jayaraj RL, Azimullah S, Beiram R, Jalal FY, Rosenberg GA (2019). Neuroinflammation: friend and foe for ischemic stroke. J Neuroinflammation.

[4] Candelario-Jalil E (2009). Injury and repair mechanisms in ischemic stroke: considerations for the development of novel neurotherapeutics. Curr Opin Investig Drugs.

[5] Candelario-Jalil E, Dijkhuizen RM, Magnus T (2022). Neuroinflammation, stroke, blood–brain barrier dysfunction, and imaging modalities. Stroke.

[6] Iadecola C, Buckwalter MS, Anrather J (2020). Immune responses to stroke: mechanisms, modulation, and therapeutic potential. J Clin Invest.

[7] Tuo QZ, Zhang ST, Lei P (2022). Mechanisms of neuronal cell death in ischemic stroke and their therapeutic implications. Med Res Rev.

[8] Stanley D, Mason LJ, Mackin KE, Srikhanta YN, Lyras D, Prakash MD, Nurgali K, Venegas A, Hill MD, Moore RJ, Wong CH (2016). Translocation and dissemination of commensal bacteria in post-stroke infection. Nat Med.

[9] Crapser J, Ritzel R, Verma R, Venna VR, Liu F, Chauhan A, Koellhoffer E, Patel A, Ricker A, Maas K (2016). Ischemic stroke induces gut permeability and enhances bacterial translocation leading to sepsis in aged mice. Aging (Albany NY).

[10] Liu Q, Johnson EM, Lam RK, Wang Q, Bo Ye H, Wilson EN, Minhas PS, Liu L, Swarovski MS, Tran S (2019). Peripheral TREM1 responses to brain and intestinal immunogens amplify stroke severity. Nat Immunol.

[11] Ahnstedt H, Patrizz A, Chauhan A, Roy-O'Reilly M, Furr JW, Spychala MS, D'Aigle J, Blixt FW, Zhu L, Bravo Alegria J, McCullough LD (2020). Sex differences in T cell immune responses, gut permeability and outcome after ischemic stroke in aged mice. Brain Behav Immun.

[12] Kurita N, Yamashiro K, Kuroki T, Tanaka R, Urabe T, Ueno Y, Miyamoto N, Takanashi M, Shimura H, Inaba T (2020). Metabolic endotoxemia promotes neuroinflammation after focal cerebral ischemia. J Cereb Blood Flow Metab.

[13] El-Hakim Y, Mani KK, Eldouh A, Pandey S, Grimaldo MT, Dabney A, Pilla R, Sohrabji F (2021). Sex differences in stroke outcome correspond to rapid and severe changes in gut permeability in adult Sprague-Dawley rats. Biol Sex Differ.

[14] Wen SW, Shim R, Ho L, Wanrooy BJ, Srikhanta YN, Prame Kumar K, Nicholls AJ, Shen SJ, Sepehrizadeh T, de Veer M (2019). Advanced age promotes colonic dysfunction and gut-derived lung infection after stroke. Aging Cell.

[15] Lee J, d'Aigle J, Atadja L, Quaicoe V, Honarpisheh P, Ganesh BP, Hassan A, Graf J, Petrosino J, Putluri N (2020). Gut microbiota-derived short-chain fatty acids promote poststroke recovery in aged mice. Circ Res.

[16] Kim YK, Shin JS, Nahm MH (2016). NOD-like receptors in infection, immunity, and diseases. Yonsei Med J.

[17] Pei G, Zyla J, He L, Moura-Alves P, Steinle H, Saikali P, Lozza L, Nieuwenhuizen N, Weiner J, Mollenkopf HJ (2021). Cellular stress promotes NOD1/2-dependent inflammation via the endogenous metabolite sphingosine-1-phosphate. EMBO J.

[18] Kim SW, Oh SA, Seol SI, Davaanyam D, Lee JK (2022). Cytosolic HMGB1 mediates LPS-induced autophagy in microglia by interacting with NOD2 and suppresses its proinflammatory function. Cells.

[19] Inohara N, del Peso L, Koseki T, Chen S, Nunez G (1998). RICK, a novel protein kinase containing a caspase recruitment domain, interacts with CLARP and regulates CD95-mediated apoptosis. J Biol Chem.

[20] McCarthy JV, Ni J, Dixit VM (1998). RIP2 is a novel NF-kappaB-activating and cell death-inducing kinase. J Biol Chem.

[21] Zhang WH, Wang X, Narayanan M, Zhang Y, Huo C, Reed JC, Friedlander RM (2003). Fundamental role of the Rip2/caspase-1 pathway in hypoxia and ischemia-induced neuronal cell death. Proc Natl Acad Sci USA.

[22] Windheim M, Lang C, Peggie M, Plater LA, Cohen P (2007). Molecular mechanisms involved in the regulation of cytokine production by muramyl dipeptide. Biochem J.

[23] White BJ, Tarabishy S, Venna VR, Manwani B, Benashski S, McCullough LD, Li J (2012). Protection from cerebral ischemia by inhibition of TGFbeta-activated kinase. Exp Neurol.

[24] Zeyen T, Noristani R, Habib S, Heinisch O, Slowik A, Huber M, Schulz JB, Reich A, Habib P (2020). Microglial-specific depletion of TAK1 is neuroprotective in the acute phase after ischemic stroke. J Mol Med (Berl).

[25] Liu Y, Li S, Wang R, Pu H, Zhao Y, Ye Q, Shi Y (2021). Inhibition of TGFbeta-activated kinase 1 promotes inflammation-resolving microglial/macrophage responses and recovery after stroke in ovariectomized female mice. Neurobiol Dis.

[26] Shaw PJ, Barr MJ, Lukens JR, McGargill MA, Chi H, Mak TW, Kanneganti TD (2011). Signaling via the RIP2 adaptor protein in central nervous system-infiltrating dendritic cells promotes inflammation and autoimmunity. Immunity.

[27] Yang C, Yang Y, DeMars KM, Rosenberg GA, Candelario-Jalil E (2020). Genetic deletion or pharmacological inhibition of cyclooxygenase-2 reduces blood–brain barrier damage in experimental ischemic stroke. Front Neurol.

[28] DeMars KM, Yang C, Candelario-Jalil E (2019). Neuroprotective effects of targeting BET proteins for degradation with dBET1 in aged mice subjected to ischemic stroke. Neurochem Int.

[29] Yang C, Lavayen BP, Liu L, Sanz BD, DeMars KM, Larochelle J, Pompilus M, Febo M, Sun YY, Kuo YM (2021). Neurovascular protection by adropin in experimental ischemic stroke through an endothelial nitric oxide synthase-dependent mechanism. Redox Biol.

[30] Trendelenburg G, Prass K, Priller J, Kapinya K, Polley A, Muselmann C, Ruscher K, Kannbley U, Schmitt AO, Castell S (2002). Serial analysis of gene expression identifies metallothionein-II as major neuroprotective gene in mouse focal cerebral ischemia. J Neurosci.

[31] Hawkins KE, DeMars KM, Singh J, Yang C, Cho HS, Frankowski JC, Dore S, Candelario-Jalil E (2014). Neurovascular protection by post-ischemic intravenous injections of the lipoxin A4 receptor agonist, BML-111, in a rat model of ischemic stroke. J Neurochem.

[32] Ge Y, Zadeh M, Mohamadzadeh M (2022). Vitamin B12 regulates the transcriptional, metabolic, and epigenetic programing in human ileal epithelial cells. Nutrients.

[33] Ge Y, Zadeh M, Mohamadzadeh M (2022). Vitamin B12 coordinates ileal epithelial cell and microbiota functions to resist Salmonella infection in mice. J Exp Med.

[34] Ge Y, Gong M, Zadeh M, Li J, Abbott JR, Li W, Morel L, Sonon R, Supekar NT, Azadi P (2020). Regulating colonic dendritic cells by commensal glycosylated large surface layer protein A to sustain gut homeostasis against pathogenic inflammation. Mucosal Immunol.

[35] Chomczynski P, Sacchi N (2006). The single-step method of RNA isolation by acid guanidinium thiocyanate-phenol-chloroform extraction: twenty-something years on. Nat Protoc.

[36] Liu L, Yang C, Lavayen BP, Tishko RJ, Larochelle J, Candelario-Jalil E (2022). Targeted BRD4 protein degradation by dBET1 ameliorates acute ischemic brain injury and improves functional outcomes associated with reduced neuroinflammation and oxidative stress and preservation of blood–brain barrier integrity. J Neuroinflammation.

[37] Bieber M, Gronewold J, Scharf AC, Schuhmann MK, Langhauser F, Hopp S, Mencl S, Geuss E, Leinweber J, Guthmann J (2019). Validity and reliability of neurological scores in mice exposed to middle cerebral artery occlusion. Stroke.

[38] Schaar KL, Brenneman MM, Savitz SI (2010). Functional assessments in the rodent stroke model. Exp Transl Stroke Med.

[39] Glushakov AV, Robbins SW, Bracy CL, Narumiya S, Dore S (2013). Prostaglandin F2alpha FP receptor antagonist improves outcomes after experimental traumatic brain injury. J Neuroinflammation.

[40] Liu L, Vollmer MK, Ahmad AS, Fernandez VM, Kim H, Dore S (2019). Pretreatment with Korean red ginseng or dimethyl fumarate attenuates reactive gliosis and confers sustained neuroprotection against cerebral hypoxic-ischemic damage by an Nrf2-dependent mechanism. Free Radic Biol Med.

[41] Tillerson JL, Caudle WM, Reveron ME, Miller GW (2002). Detection of behavioral impairments correlated to neurochemical deficits in mice treated with moderate doses of 1-methyl-4-phenyl-1,2,3,6-tetrahydropyridine. Exp Neurol.

[42] Kim ST, Son HJ, Choi JH, Ji IJ, Hwang O (2010). Vertical grid test and modified horizontal grid test are sensitive methods for evaluating motor dysfunctions in the MPTP mouse model of Parkinson's disease. Brain Res.

[43] Deacon RM (2013). Measuring the strength of mice. J Vis Exp.

[44] Yang C, Liu L, Lavayen BP, Larochelle J, Gunraj RE, Butler AA, Candelario-Jalil E (2023). Therapeutic benefits of adropin in aged mice after transient ischemic stroke via reduction of blood–brain barrier damage. Stroke.

[45] Ito D, Imai Y, Ohsawa K, Nakajima K, Fukuuchi Y, Kohsaka S (1998). Microglia-specific localisation of a novel calcium binding protein, Iba1. Brain Res Mol Brain Res.

[46] Sasaki Y, Ohsawa K, Kanazawa H, Kohsaka S, Imai Y (2001). Iba1 is an actin-cross-linking protein in macrophages/microglia. Biochem Biophys Res Commun.

[47] Carson RG (2018). Get a grip: individual variations in grip strength are a marker of brain health. Neurobiol Aging.

[48] Bohannon RW (2019). Grip strength: an indispensable biomarker for older adults. Clin Interv Aging.

[49] Ekstrand E, Lexell J, Brogardh C (2016). Grip strength is a representative measure of muscle weakness in the upper extremity after stroke. Top Stroke Rehabil.

[50] El Husseini N, Katzan IL, Rost NS, Blake ML, Byun E, Pendlebury ST, Aparicio HJ, Marquine MJ, Gottesman RF, Smith EE (2023). Cognitive impairment after ischemic and hemorrhagic stroke: a scientific statement from the American Heart Association/American Stroke Association. Stroke.

[51] Shimada K, Chen S, Dempsey PW, Sorrentino R, Alsabeh R, Slepenkin AV, Peterson E, Doherty TM, Underhill D, Crother TR, Arditi M (2009). The NOD/RIP2 pathway is essential for host defenses against *Chlamydophila pneumoniae* lung infection. PLoS Pathog.

[52] Jeong YJ, Kang MJ, Lee SJ, Kim CH, Kim JC, Kim TH, Kim DJ, Kim D, Nunez G, Park JH (2014). Nod2 and Rip2 contribute to innate immune responses in mouse neutrophils. Immunology.

[53] Jun JC, Cominelli F, Abbott DW (2013). RIP2 activity in inflammatory disease and implications for novel therapeutics. J Leukoc Biol.

[54] Hofmann SR, Girschick L, Stein R, Schulze F (2021). Immune modulating effects of receptor interacting protein 2 (RIP2) in autoinflammation and immunity. Clin Immunol.

[55] Honjo H, Watanabe T, Kamata K, Minaga K, Kudo M (2021). RIPK2 as a new therapeutic target in inflammatory bowel diseases. Front Pharmacol.

[56] Tigno-Aranjuez JT, Benderitter P, Rombouts F, Deroose F, Bai X, Mattioli B, Cominelli F, Pizarro TT, Hoflack J, Abbott DW (2014). In vivo inhibition of RIPK2 kinase alleviates inflammatory disease. J Biol Chem.

[57] Nachbur U, Stafford CA, Bankovacki A, Zhan Y, Lindqvist LM, Fiil BK, Khakham Y, Ko HJ, Sandow JJ, Falk H (2015). A RIPK2 inhibitor delays NOD signalling events yet prevents inflammatory cytokine production. Nat Commun.

[58] Salla M, Aguayo-Ortiz R, Danmaliki GI, Zare A, Said A, Moore J, Pandya V, Manaloor R, Fong S, Blankstein AR (2018). Identification and characterization of novel receptor-interacting serine/threonine-protein kinase 2 inhibitors using structural similarity analysis. J Pharmacol Exp Ther.

[59] Ermann J, Matmusaev M, Haley EK, Braun C, Jost F, Mayer-Wrangowski S, Hsiao P, Ting N, Li L, Terenzio D (2021). The potent and selective RIPK2 inhibitor BI 706039 improves intestinal inflammation in the TRUC mouse model of inflammatory bowel disease. Am J Physiol Gastrointest Liver Physiol.

[60] Yuan X, Chen Y, Tang M, Wei Y, Shi M, Yang Y, Zhou Y, Yang T, Liu J, Liu K (2022). Discovery of potent and selective receptor-interacting serine/threonine protein kinase 2 (RIPK2) inhibitors for the treatment of inflammatory bowel diseases (IBDs). J Med Chem.

[61] Lai Y, Wang X, Sun X, Wu S, Chen X, Yang C, Zhang W, Yu X, Tong Y, Ma F (2023). Discovery of a novel RIPK2 inhibitor for the treatment of inflammatory bowel disease. Biochem Pharmacol.

[62] Doherty MK, Hammond DE, Clague MJ, Gaskell SJ, Beynon RJ (2009). Turnover of the human proteome: determination of protein intracellular stability by dynamic SILAC. J Proteome Res.

[63] Bondeson DP, Mares A, Smith IE, Ko E, Campos S, Miah AH, Mulholland KE, Routly N, Buckley DL, Gustafson JL (2015). Catalytic in vivo protein knockdown by small-molecule PROTACs. Nat Chem Biol.

[64] Mares A, Miah AH, Smith IED, Rackham M, Thawani AR, Cryan J, Haile PA, Votta BJ, Beal AM, Capriotti C (2020). Extended pharmacodynamic responses observed upon PROTAC-mediated degradation of RIPK2. Commun Biol.

[65] Zhang X, Ramirez CM, Aryal B, Madrigal-Matute J, Liu X, Diaz A, Torrecilla-Parra M, Suarez Y, Cuervo AM, Sessa WC, Fernandez-Hernando C (2020). Cav-1 (Caveolin-1) deficiency increases autophagy in the endothelium and attenuates vascular inflammation and atherosclerosis. Arterioscler Thromb Vasc Biol.

[66] Zhang X, Gong P, Zhao Y, Wan T, Yuan K, Xiong Y, Wu M, Zha M, Li Y, Jiang T (2022). Endothelial caveolin-1 regulates cerebral thrombo-inflammation in acute ischemia/reperfusion injury. EBioMedicine.

[67] He R, Yuan X, Lv X, Liu Q, Tao L, Meng J (2022). Caveolin-1 negatively regulates inflammation and fibrosis in silicosis. J Cell Mol Med.

[68] Huang Q, Zhong W, Hu Z, Tang X (2018). A review of the role of cav-1 in neuropathology and neural recovery after ischemic stroke. J Neuroinflammation.

[69] Gu Y, Zheng G, Xu M, Li Y, Chen X, Zhu W, Tong Y, Chung SK, Liu KJ, Shen J (2012). Caveolin-1 regulates nitric oxide-mediated matrix metalloproteinases activity and blood-brain barrier permeability in focal cerebral ischemia and reperfusion injury. J Neurochem.

[70] Bi F, Huang C, Tong J, Qiu G, Huang B, Wu Q, Li F, Xu Z, Bowser R, Xia XG, Zhou H (2013). Reactive astrocytes secrete lcn2 to promote neuron death. Proc Natl Acad Sci USA.

[71] Luo C, Zhou S, Yin S, Jian L, Luo P, Dong J, Liu E (2022). Lipocalin-2 and cerebral stroke. Front Mol Neurosci.

