# Supplementary material for: Receptor-interacting protein kinase 2 (RIPK2) profoundly contributes to post-stroke neuroinflammation and behavioral deficits with microglia as unique perpetrators
Source: J Neuroinflammation. 2023 Sep 30;20:221. doi: 10.1186/s12974-023-02907-6 (PMC10543871; doi:10.1186/s12974-023-02907-6)
Supplement: Supplementary file 1 — Additional file 1: Ripk2−/− mice show no differences in cerebral vessel parameters at baseline or cerebral blood flow during stroke surgery. A Representative vessel casting images depicting the superior (top) and inferior (bottom) views of Ripk2−/− and Ripk2+/+ following latex vessel casting. Square inserts depict method for measuring the distance from baseline and for counting the number of anastomoses. B The number of anastomotic connections between vessels originating from the brain midline to the branches of the middle cerebral artery. C The distance from anastomotic connections to the brain midline. D The diameter of various cerebral arteries. E Cerebral blood flow measured during tMCAO surgery for both genotypes. (A–D) n = 4–5/group. Differences determined by Student’s t test (B) or two-way ANOVA (C, D). Abbreviations: ACA: anterior cerebral artery, MCA: middle cerebral artery, ICA: internal carotid artery, PCA: posterior cerebral artery. [file 12974_2023_2907_MOESM1_ESM.pdf]

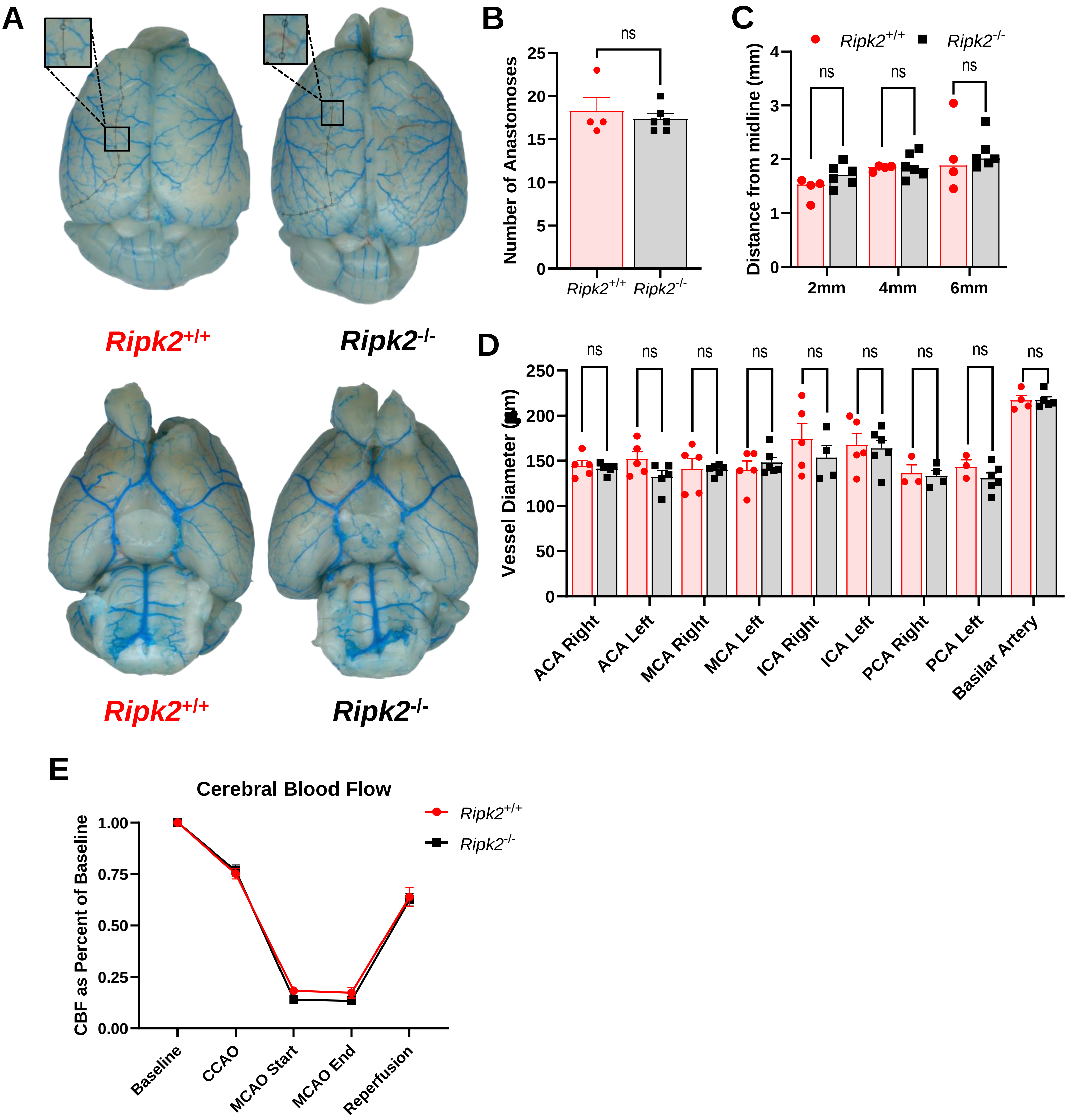
A
Ripk2+/+
Ripk2-/-
Ripk2+/+
Ripk2-/-
B
ns
Number of Anastomoses
0
5
10
15
20
25
Ripk2+/+
Ripk2-/-
C
Ripk2+/+
Ripk2-/-
Distance from midline (mm)
0
1
2
3
4
ns
ns
ns
2mm
4mm
6mm
D
Vessel Diameter (µm)
0
50
100
150
200
250
ns
ACA Right
ACA Left
MCA Right
MCA Left
ICA Right
ICA Left
PCA Right
PCA Left
Basilar Artery
E
Cerebral Blood Flow
CBF as Percent of Baseline
0.00
0.25
0.50
0.75
1.00
Ripk2+/+
Ripk2-/-
Baseline
CCAO
MCAO Start
MCAO End
Reperfusion

**Additional File 1**: *Ripk2*$^{-/-}$ mice show no differences in cerebral vessel parameters at baseline or cerebral blood flow during stroke surgery. **A** Representative vessel casting images depicting the superior (top) and inferior (bottom) views of *Ripk2*$^{-/-}$ and *Ripk2*$^{+/+}$ following latex vessel casting. Square inserts depict method for measuring the distance from baseline and for counting the number of anastomoses. **B** The number of anastomotic connections between vessels originating from the brain midline to the branches of the middle cerebral artery. **C** The distance from anastomotic connections to the brain midline. **D** The diameter of various cerebral arteries. **E** Cerebral blood flow measured during tMCAO surgery for both genotypes. (A-D) n=4-5/group. Differences determined by Student's t test (B) or two-way ANOVA (C-D). Abbreviations: ACA: anterior cerebral artery, MCA: middle cerebral artery, ICA: internal carotid artery, PCA: posterior cerebral artery.
