# Supplementary material for: Receptor-interacting protein kinase 2 (RIPK2) profoundly contributes to post-stroke neuroinflammation and behavioral deficits with microglia as unique perpetrators
Source: J Neuroinflammation. 2023 Sep 30;20:221. doi: 10.1186/s12974-023-02907-6 (PMC10543871; doi:10.1186/s12974-023-02907-6)
Supplement: Supplementary file 2 — Additional file 2: Differences in neurological deficit score parameters between Ripk2-/- and Ripk2+/+ mice during the 28-day longitudinal study. A–E Neurological deficit scores for each of the five parameters, with total scores for each indicated timepoint. Scores were recorded at 24 h (A), 48 h (B), 7d (C), 14d (D), and 21d (E) post-stroke. n = 14–21 per group at 24 h and 48 h, n = 11–14/genotype at 7d, n = 10–14/genotype at 14d and 21d. Differences determined by Mann–Whitney test. * P < 0.05, ** P < 0.01 *** P < 0.001 **** P < 0.0001 [file 12974_2023_2907_MOESM2_ESM.pdf]

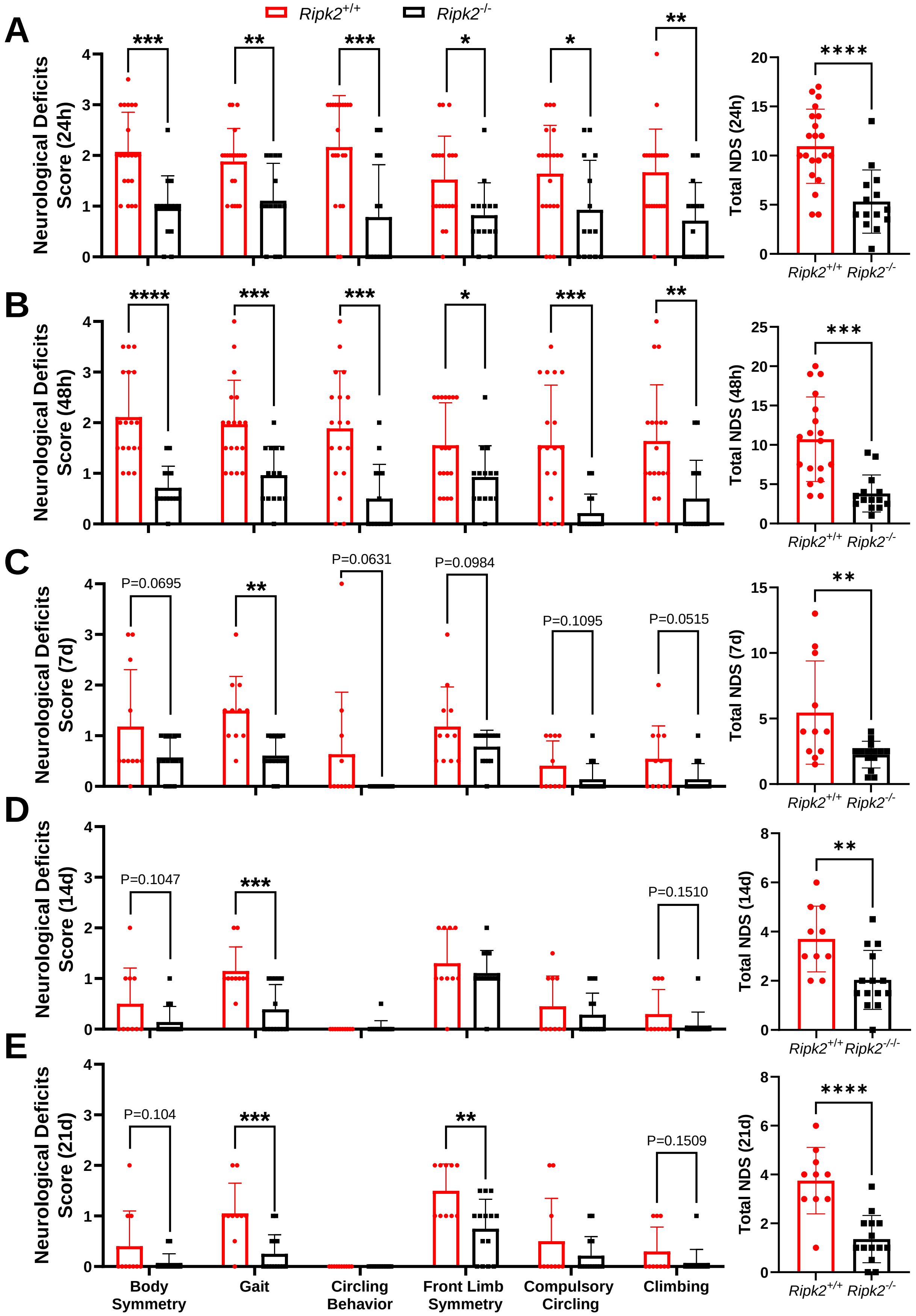

Ripk2+/+
Ripk2-/-
A
Neurological Deficits Score (24h)
***
**
***
*
*
**
Total NDS (24h)
****
B
Neurological Deficits Score (48h)
****
***
***
*
***
**
Total NDS (48h)
***
C
Neurological Deficits Score (7d)
P=0.0695
**
P=0.0631
P=0.0984
P=0.1095
P=0.0515
Total NDS (7d)
**
D
Neurological Deficits Score (14d)
P=0.1047
***
P=0.1510
Total NDS (14d)
**
E
Neurological Deficits Score (21d)
P=0.104
***
**
P=0.1509
Total NDS (21d)
****
Body Symmetry
Gait
Circling Behavior
Front Limb Symmetry
Compulsory Circling
Climbing

Additional File 2: Differences in neurological deficit score parameters between *Ripk2*$^{-/-}$ and *Ripk2*$^{+/+}$ mice during the 28-day longitudinal study. **A-E** Neurological deficit scores for each of the five parameters, with total scores for each indicated timepoint. Scores were recorded at 24h (A), 48h (B), 7d (C), 14d (D), and 21d (E) post-stroke. n=14-21 per group at 24h and 48h, n=11-14/genotype at 7d, n=10-14/genotype at 14d and 21d. Differences determined by Mann-Whitney test. * P<0.05, ** P<0.01 *** P<0.001 **** P<0.0001
