# Supplementary material for: Receptor-interacting protein kinase 2 (RIPK2) profoundly contributes to post-stroke neuroinflammation and behavioral deficits with microglia as unique perpetrators
Source: J Neuroinflammation. 2023 Sep 30;20:221. doi: 10.1186/s12974-023-02907-6 (PMC10543871; doi:10.1186/s12974-023-02907-6)
Supplement: Supplementary file 3 — Additional file 3: No differences in the time spent in the center of the open field chamber between genotypes. Recordings for the time mice spent in the center of the open field chamber were taken at baseline then at 48 h, 7-day, 14-day, and 21-day post-stroke. n = 10–14mice/group. No differences were determined via two-way ANOVA, multiple comparisons. [file 12974_2023_2907_MOESM3_ESM.pdf]

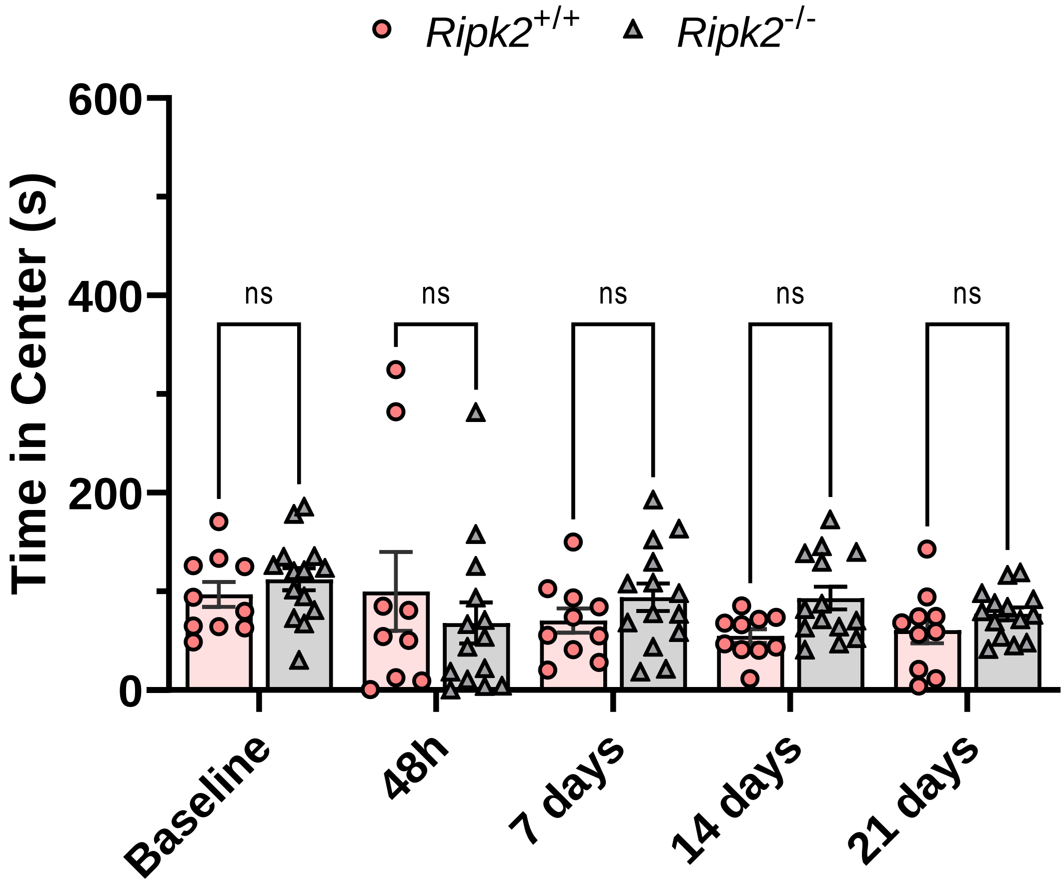

Additional File 3: No differences in the time spent in the center of the open field chamber between genotypes. Recordings for the time mice spent in the center of the open field chamber were taken at baseline then at 48h, 7 days, 14 days, and 21 days post-stroke. n=10-14mice/group. No differences were determined via two-way ANOVA, multiple comparisons.
