# Supplementary material for: Receptor-interacting protein kinase 2 (RIPK2) profoundly contributes to post-stroke neuroinflammation and behavioral deficits with microglia as unique perpetrators
Source: J Neuroinflammation. 2023 Sep 30;20:221. doi: 10.1186/s12974-023-02907-6 (PMC10543871; doi:10.1186/s12974-023-02907-6)
Supplement: Supplementary file 4 — Additional file 4: Ripk2−/− mice experience similar infarct volumes with 60min of occlusion compared to Ripk2+/+ with 45min of occlusion. To correct for differences in infarct volume before performing our bulk RNAseq on microglia, the Ripk2−/− mice were subjected to an additional 15 min of occlusion time compared to Ripk2+/+ mice. This produced a similar level of infarction between the two genotypes. n = 5–10mice/group. No differences determined by Student’s t test. [file 12974_2023_2907_MOESM4_ESM.pdf]

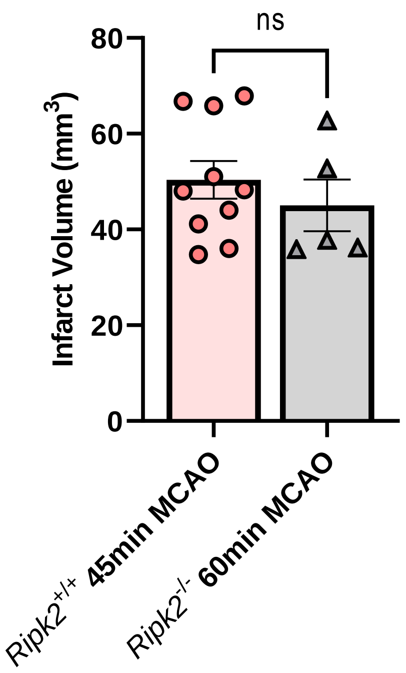

Additional File 4: *Ripk2*$^{-/-}$ mice experience similar infarct volumes with 60min of occlusion compared to *Ripk2*$^{+/+}$ with 45min of occlusion. To correct for differences in infarct volume before performing our bulk RNAseq on microglia, the *Ripk2*$^{-/-}$ mice were subjected to an additional 15min of occlusion time compared to *Ripk2*$^{+/+}$ mice. This produced a similar level of infarction between the two genotypes. n=5-10mice/group. No differences determined by Student’s t test.
