# Supplementary material for: Receptor-interacting protein kinase 2 (RIPK2) profoundly contributes to post-stroke neuroinflammation and behavioral deficits with microglia as unique perpetrators
Source: J Neuroinflammation. 2023 Sep 30;20:221. doi: 10.1186/s12974-023-02907-6 (PMC10543871; doi:10.1186/s12974-023-02907-6)
Supplement: Supplementary file 5 — Additional file 5 A: No observed differences in cerebral blood flow during the course of tMCAO surgery in WT vs μKO mice. B No statistical differences in survival during the 48h period following stroke induction; however, μKO mice suffered 1 animal loss compared to 3 in the WT group. n=13/genotype. [file 12974_2023_2907_MOESM5_ESM.pdf]

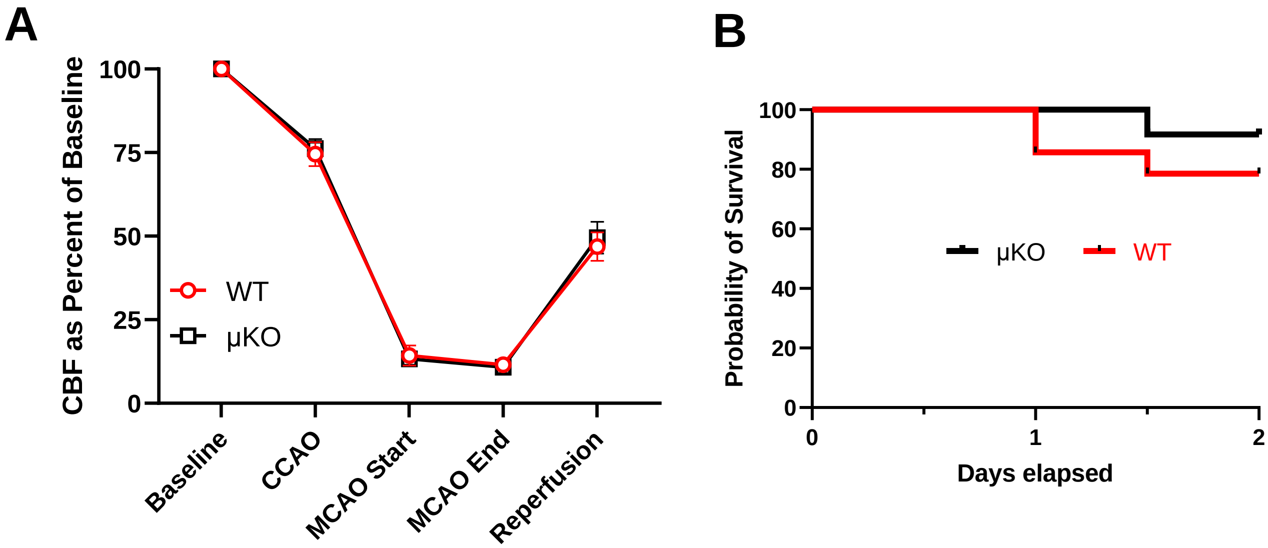

Additional File 5: **A** No observed differences in cerebral blood flow during the course of tMCAO surgery in WT vs μKO mice. **B** No statistical differences in survival during the 48h period following stroke induction, however μKO mice suffered 1 animal loss compared to 3 in the WT group. n=13/genotype.
