# Supplementary figures and images for: Receptor-interacting protein kinase 2 (RIPK2) profoundly contributes to post-stroke neuroinflammation and behavioral deficits with microglia as unique perpetrators
Source: J Neuroinflammation. 2023 Sep 30;20:221. doi: 10.1186/s12974-023-02907-6 (PMC10543871; doi:10.1186/s12974-023-02907-6)

# Fig1A

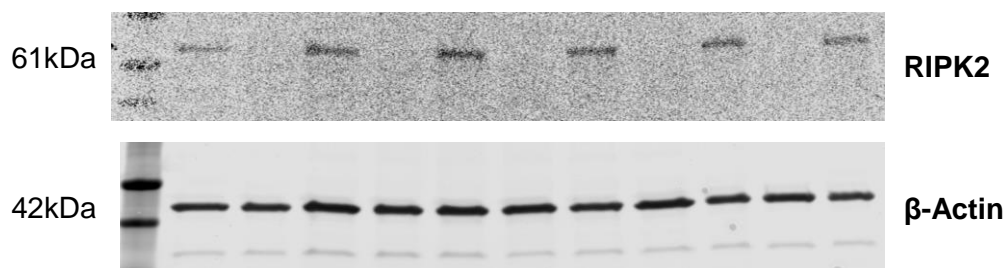

# Fig2C

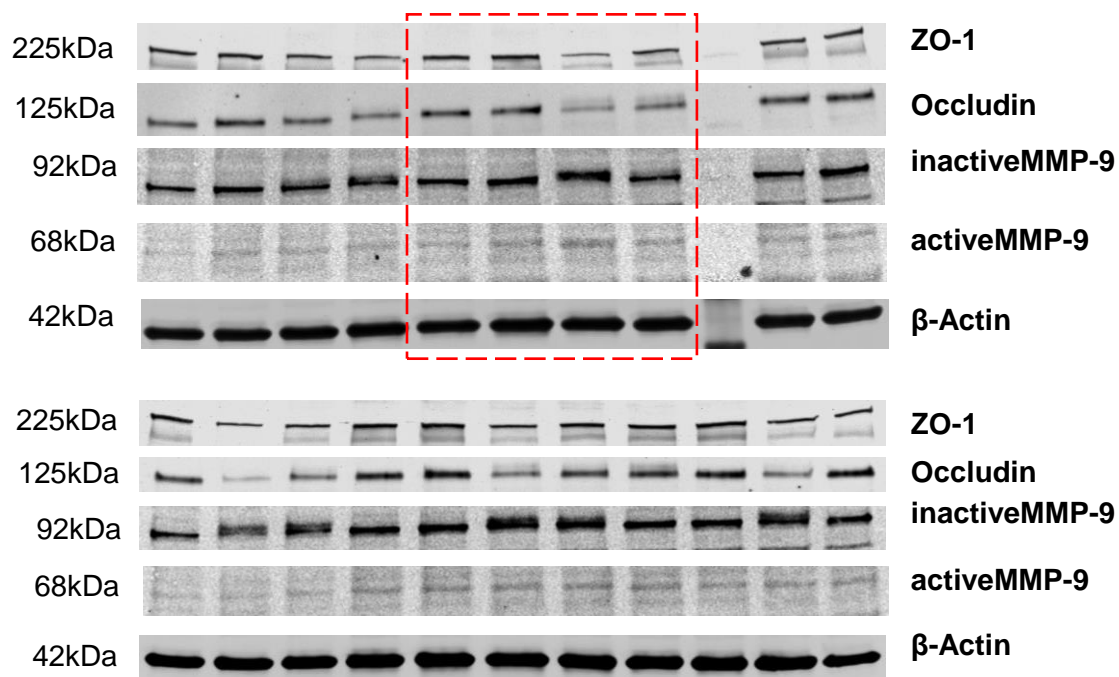

# Fig8I

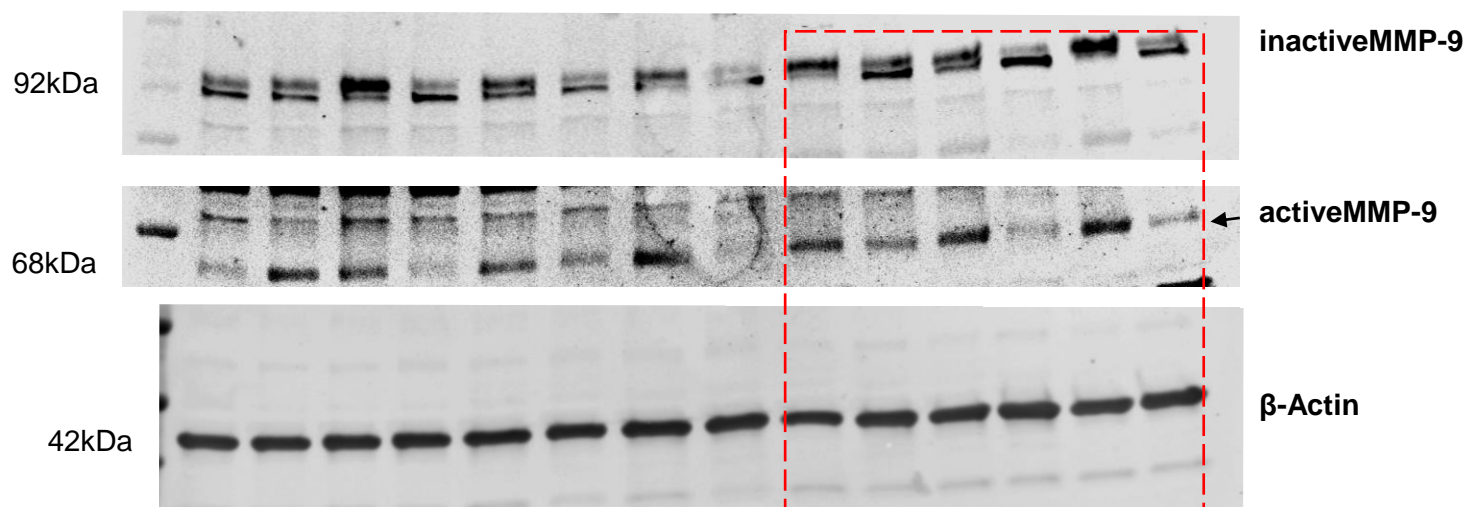

Supplement: Supplementary file 6 — Additional file 6: Unedited Western blots. [file 12974_2023_2907_MOESM6_ESM.pdf]
